# Dietary theabrownins alleviate age-related declines in productive performance and egg quality by improving intestinal health in laying hens

**DOI:** 10.1186/s40104-026-01480-1

**Published:** 2026-08-10

**Authors:** Li Zhang, Wenwen Xu, Keying Zhang, Xuemei Ding, Qiufeng Zeng, Shiping Bai, Jingbo Liu, Jianping Wang

**Affiliations:** 1 Key Laboratory of Animal Disease-Resistance Nutrition, Ministry of Education, Ministry of Agriculture and Rural Affairs, Key Laboratory of Sichuan Province, Animal Nutrition Institute, Sichuan Agricultural University, Chengdu, China; 2 State Key Laboratory of Animal Nutrition and Feeding, Institute of Animal Science, Chinese Academy of Agricultural Sciences, Beijing, 100193 People’s Republic of China; 3 College of Life Sciences and Agri-Forestry, Southwest University of Science and Technology, Mianyang, 621010 People’s Republic of China

**Keywords:** Aging, Cecal microbiota, Intestinal morphology, Laying hens, Theabrownins

## Abstract

**Background:**

The late laying period is often accompanied by declines in productive performance, egg quality, and intestinal health in hens. This study used a 2 × 2 factorial design to evaluate the effects of dietary theabrownins (TB), bioactive polyphenols derived from dark tea, on production performance, egg quality, intestinal health, and cecal microbiota in 47- and 67-week-old laying hens. A total of 192 Lohmann Gray hens were fed diets containing 0 or 100 mg/kg TB for 12 weeks.

**Results:**

Significant age × TB interactions were observed for average daily feed intake (ADFI; *P* = 0.005), eggshell thickness (*P* = 0.001), jejunal villus height (*P* = 0.005), and *TJP2* expression (*P* = 0.029). Compared with 47-week-old hens, 67-week-old hens had higher egg weight, feed-to-egg ratio, and unqualified egg rate, but lower laying rate, egg mass, Haugh unit, and eggshell strength (all *P* < 0.01). TB supplementation increased laying rate (*P* < 0.001), egg mass (*P* = 0.003), Haugh unit (*P* = 0.015), eggshell strength (*P* = 0.010), and albumen height (*P* = 0.018), while reducing feed-to-egg ratio (*P* = 0.003). TB also improved jejunal morphology by increasing villus height and reducing crypt depth, enhanced antioxidant capacity by increasing T-AOC (*P* = 0.002) and GST activity (*P* = 0.025), and upregulated jejunal *TJP2* expression in older hens. In addition, TB decreased the Firmicutes/Bacteroidota ratio (*P* < 0.001) and enriched *Prevotellaceae_UCG-001* abundance. Notably, correlation analysis showed that *Prevotellaceae_UCG-001* was positively correlated with intestinal antioxidant indices, whereas productive and egg quality traits were significantly associated with intestinal structural and functional indicators.

**Conclusion:**

Dietary TB supplementation partly alleviated age-related declines in productive performance and egg quality in laying hens, possibly through improving intestinal health and modulating cecal microbiota. Notably, the TB-enriched genus *Prevotellaceae_UCG-001* was positively associated with intestinal antioxidant indices, suggesting a potential microbial link to the intestinal benefits of TB in aged laying hens. These findings support the potential application of TB as a functional feed additive to help maintain intestinal health and productive performance in aged laying hens.

**Graphical Abstract:**

**Figure:**
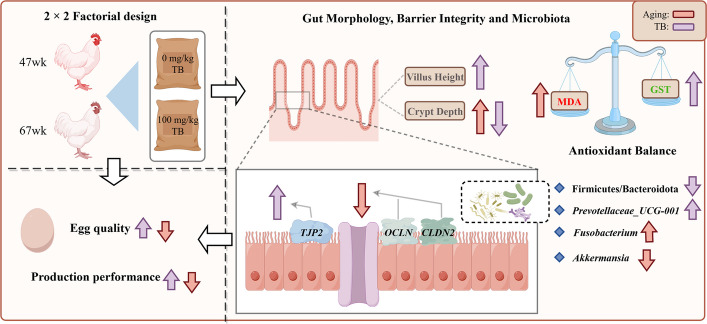

**Supplementary Information:**

The online version contains supplementary material available at 10.1186/s40104-026-01480-1.

## Introduction

Eggs are a widely consumed and nutritionally valuable food product that provides high-quality protein, essential fatty acids, vitamins, and minerals. As an accessible source of animal protein, eggs contribute significantly to human health and help reduce nutrient deficiencies in diverse populations [1, 2]. The global demand for eggs continues to grow, highlighting the importance of maintaining optimal egg production in laying hens throughout their productive lifespan. During the early and middle stages of the laying cycle, egg production typically increases with the age of hens. However, as hens enter the late laying phase, physiological aging processes are accompanied by progressive declines in productive performance, feed efficiency, and health status [3–5]. In addition, deterioration in egg quality during the late laying period further reduces the economic value of aged hens.

Aging is closely associated with intestinal structural and functional deterioration, which impairs nutrient absorption, immune regulation, and metabolic stability [6]. In particular, intestinal villus morphology is a key indicator of gut health, and segment-specific differences in villus structure are closely related to intestinal function, with the duodenum mainly involved in digestion, the jejunum in nutrient absorption, and the ileum in immune regulation [7]. These structures undergo continuous renewal and are influenced by age, diet, and microbial composition. Tight junction proteins are critical components of the epithelial barrier and play essential roles in maintaining intestinal integrity and regulating paracellular permeability. Among these, tight junction protein 1 (*TJP1*), tight junction protein 2 (*TJP2*), occludin (*OCLN*), and claudin 2 (*CLDN2*) are commonly associated with epithelial barrier structure and permeability regulation [8, 9]. In addition, mucin 2 (*MUC2*) is a major component of the intestinal mucus layer and contributes to mucosal protection [10]. Aging-related intestinal dysfunction is often accompanied by low-grade inflammation, in which proinflammatory cytokines, such as interleukin 6 (*IL-6*), interleukin 1 beta (*IL-1β*), and tumor necrosis factor (*TNF*), are involved [11]. Additionally, age-associated dysbiosis in the cecum impairs short-chain fatty acid (SCFA) production and compromises barrier function. Declines in beneficial taxa such as *Lactobacillus* and *Akkermansia* may aggravate inflammation and oxidative stress, thereby accelerating intestinal aging [12]. Given the central role of the intestine in nutrient digestion and utilization, age-related intestinal dysfunction is likely to be an important factor contributing to the decline in productive performance and egg quality in late-laying hens.

Improving intestinal health in aging laying hens is therefore regarded as a promising strategy to support sustained production and extend the laying period. Dietary supplementation with functional bioactive compounds has attracted increasing attention as a non-antibiotic approach to promote gut health [13–16]. Among various bioactive compounds derived from natural sources, theabrownins (TB) have emerged as promising candidates for improving host health and productivity. TB are high-molecular-weight polyphenolic pigments formed during the microbial fermentation and oxidation processes of dark tea, especially Pu-erh tea [17, 18]. Unlike monomeric catechins or theaflavins found in green and black teas, TB are complex polymeric structures with stable biological activity and prolonged retention in the gastrointestinal tract, allowing for extended physiological effects [19]. Accumulating evidence suggests that TB possess a range of beneficial properties, most notably antioxidant, anti-inflammatory, and gut microbiota-modulating functions [20–22]. For instance, Wang et al. [23] demonstrated that dietary supplementation with 400 mg/kg TB significantly enhanced antioxidant capacity and upregulated *Nrf2*-mediated gene expression in the magnum tissue of laying hens, while reducing MDA levels and the expression of pro-apoptotic genes. In addition to their redox-modulating effects, TB have also been shown to influence gut microbial ecology, increasing the abundance of beneficial taxa such as *Lactobacillus* and *Akkermansia*, which are associated with improved intestinal health [24, 25]. Moreover, our previous study found that dietary supplementation with TB significantly improved laying performance and egg quality in aging hens by enhancing ovarian antioxidant capacity, alleviating inflammation, maintaining structural integrity of the ovary, and upregulating key genes related to follicular development and reproductive hormone synthesis [26]. These findings indicate that TB may help maintain productive function during the late laying period. However, whether such benefits are also mediated through improvements in intestinal health, another key physiological basis for sustained productivity in aged laying hens, remains unclear.

Therefore, an important unanswered question is whether dietary TB supplementation can alleviate age-related declines in productive performance and egg quality by improving intestinal health in laying hens. To address this question, the present study used a 2 × 2 factorial design in 47- and 67-week-old laying hens to evaluate the effects of TB supplementation on production performance, egg quality, intestinal histomorphology, antioxidant status, barrier-related gene expression, and cecal microbiota composition. By comparing hens at two physiological stages, this study aimed not only to assess the intestinal regulatory effects of TB supplementation, but also to determine whether its benefits are more pronounced under age-related physiological decline. These findings may provide new evidence for the application of TB as a functional feed additive to maintain intestinal health and productive longevity in aged laying hens.

## Materials and methods

### Animals and experimental design

A 2 × 2 factorial arrangement was employed to investigate the effects of dietary TB supplementation on Lohmann Gray laying hens at two physiological stages: 47 and 67 weeks of age. A total of 192 clinically healthy hens (initial body weight: 1.58 ± 0.12 kg), all originating from a common parental stock. Two dietary treatments were formulated: a control diet (0 mg/kg TB) and a TB-supplemented diet (100 mg/kg TB). Within each age group, 96 hens were randomly allocated to one of the two dietary regimens. Each treatment group comprised 8 replicates, each consisting of 6 birds housed in two adjacent cages (3 birds per cage). Birds were maintained under standardized environmental conditions and provided ad libitum access to a nutritionally adequate basal diet formulated according to standard requirements (see Table 1 for composition). The duration of the experimental period was 12 weeks. The TB used in the trial were procured from Yunnan Tangren Biotechnology Co., Ltd. (Yunnan, China), with an assay-confirmed purity of 81%.

**Table 1:** Composition and nutrient level of basal diet (as-fed basis)

Item, %	Content
Corn	56.40
Soybean meal (43%)	25.50
Wheat bran	4.80
Soybean oil	2.34
Calcium carbonate	8.30
Calcium hydrophosphate	1.50
L-Lysine hydrochloride	0.04
DL-Methionine	0.14
Threonine	0.04
Tryptophan	0.01
NaCl	0.30
Choline chloride (50%)	0.10
Vitamin and mineral premix^a^	0.53
Total	100.00
Analyzed nutrient levels, %	
ME^b^, kcal/kg	2,703.08
Crude protein	16.58
Calcium	3.55
Available phosphorus	0.38
Lysine	0.79
Methionine	0.36
Threonine	0.58
Tryptophan	0.18
Methionine + cysteine	0.59

^a^Provided per kilogram of diet: vitamin A, 10,000 IU; vitamin D_3_, 3,000 IU; vitamin E, 30 IU; vitamin K_3_, 4.8 mg; thiamin, 3.0 mg; riboflavin, 9.6 mg; pyridoxine, 6 mg; vitamin B_12_, 0.3 mg; folic acid, 1.5 mg; niacin, 60 mg; pantothenic acid, 18 mg; biotin,0.6 mg; iron, 60 mg; copper, 8 mg; manganese, 60 mg; zinc, 80 mg; selenium, 0.30 mg; iodine, 0.35 mg

^b^Calculated according to NRC (1994) [27]

### Animal management

The experimental basal diet formulation followed the guidelines of NRC (1994) [27] and the Chinese Chicken Feeding Standard (2004) [28], with corn and soybean meal constituted the major components. The feed was provided in powdered form, with an average particle size of approximately 4.5 mm. All hens were housed in a stacked, multi-tier cage system (dimensions: 45 cm × 45 cm × 43 cm), with three birds allocated per cage. For each treatment group, two adjacent cages were used, and all birds were consistently housed at the middle tier to minimize environmental variation associated with cage height. Ambient temperature was controlled at 20 to 22 °C during the experimental period. A controlled photoperiod of 16 h light and 8 h dark (16L:8D) was implemented. Ventilation was kept uniform across all units, and routine hygiene was ensured through weekly disinfection to maintain optimal sanitary conditions.

### Production performance

Production performance was recorded throughout the 12-week experimental period on a replicate basis. Feed intake, egg number, egg weight, and unqualified eggs were recorded daily. Laying rate, average daily feed intake (ADFI), feed-to-egg ratio, egg mass, and unqualified egg rate were subsequently calculated. Laying rate was expressed as the percentage of eggs produced relative to the number of hens. ADFI was calculated as the average daily feed intake per hen. Egg mass was calculated from laying rate and average egg weight. Feed-to-egg ratio was expressed as feed intake per unit of egg mass. Unqualified egg rate was calculated as the percentage of unqualified eggs relative to total egg production.

### Egg quality assessment

At the end of the 12-week experimental period, three freshly laid eggs were randomly collected from each replicate for egg quality evaluation. Thus, 24 eggs were analyzed per treatment group, with a total of 96 eggs used for egg quality assessment. Each egg was treated as an individual biological replicate. Before analysis, the eggs were allowed to equilibrate to room temperature. The evaluated egg quality traits included egg weight, albumen height, Haugh unit, yolk color, eggshell breaking strength, and eggshell thickness. Egg weight was measured using a precision digital balance (FA1604N, Shanghai Precision Scientific Instruments, Shanghai, China). Albumen height, Haugh unit, and yolk color were determined using an Egg Multi-tester (EMT-5200, Robotmation Co., Ltd., Tokyo, Japan). Eggshell breaking strength was measured with an eggshell force gauge (Model II, Robotmation Co., Ltd., Tokyo, Japan). For eggshell thickness determination, eggs were broken manually and the shells were separated from the albumen and yolk. After removal of the shell membranes and any adhering residues, eggshell thickness was measured using a Peacock dial gauge (P-1, Ozaki MFG Co., Ltd., Tokyo, Japan) at three standardized locations: the blunt end, the sharp end, and the equatorial region. The mean of these three measurements was used as the final eggshell thickness value for each egg. All measurements were performed according to standardized operating procedures, and all instruments were calibrated prior to use to ensure the accuracy and consistency of the measurements.

### Sample collection

Following the 12-week feeding period, one bird was randomly chosen from each replicate (*n* = 8 per group) and euthanized via carbon dioxide (CO_2_) inhalation. Immediately post-mortem, the abdominal cavity was opened to expose the gastrointestinal tract. Tissue sections (~2 cm in length) were excised from the midpoint of the duodenum, jejunum, and ileum for histological assessment. Each sample was gently rinsed with sterile phosphate-buffered saline (PBS) to remove luminal contents and subsequently preserved in 10% neutral-buffered formalin for histology. Morphological parameters, including villus height and crypt depth, were evaluated using a digital microscope system (BA400Digital, McAudi Industrial Group Co., Ltd., Chengdu, China) at 40 × magnification. For gene expression analysis, an additional jejunal segment (~2 cm) was collected, flushed with PBS, rapidly frozen in liquid nitrogen, and stored at –80 °C. Cecal digesta were aseptically harvested from the distal end of the cecum using sterile tools, transferred to cryogenic tubes, snap-frozen in liquid nitrogen, and preserved at −80 °C for subsequent microbiota profiling.

### Morphology of intestinal mucosa

Duodenal, jejunal, and ileal segments fixed in 10% neutral-buffered formalin were subjected to routine histological processing, including dehydration through a graded ethanol series, clearance in xylene, and embedding in paraffin wax. Tissue blocks were sectioned at a thickness of 5 μm, stained with hematoxylin and eosin (H&E), and examined using a digital microscope with an integrated imaging system (BA400Digital, McAudi Industrial Group Co., Ltd., China) under 40 × magnification. For each intestinal region, ten intact villus–crypt units were randomly selected per sample for morphometric analysis. Villus height was determined by measuring from the tip of the villus to the villus–crypt junction, while crypt depth was defined as the distance between the base of adjacent villi. The villus height-to-crypt depth ratio was subsequently calculated to assess mucosal structural integrity.

### Determination of enzyme activity in jejunum

Jejunal tissue samples were assessed for antioxidant capacity and oxidative stress biomarkers, including total antioxidant capacity (T-AOC), glutathione (GSH), glutathione *S*-transferase (GST), catalase (CAT), glutathione peroxidase (GSH-Px), malondialdehyde (MDA), and superoxide dismutase (SOD), using commercial ELISA assay kits purchased from Nanjing Jiancheng Bioengineering Institute (Nanjing, China).

### Quantitative real-time PCR analysis of jejunal mRNA expression

Total RNA was extracted from approximately 100 mg of jejunal tissue using TRIzol® reagent (TaKaRa Biotechnology Co., Ltd., Dalian, China) in accordance with the manufacturer’s protocol. RNA concentration and purity were determined using a NanoDrop 2000 spectrophotometer (Thermo Fisher Scientific, Waltham, MA, USA), and only samples with an A_260_/A_280_ ratio between 1.8 and 2.0 were included for downstream analysis. cDNA was synthesized from 1 μg of total RNA using the PrimeScript™ RT Reagent Kit with gDNA Eraser (Cat# RR047A, TaKaRa Biotechnology Co., Ltd., Dalian, China). Quantitative real-time PCR (qPCR) was conducted using the SYBR^®^ Premix Ex Taq™ II Kit (Cat# RR820A, TaKaRa Biotechnology Co., Ltd., Dalian, China) on a StepOnePlus™ Real-Time PCR System (Applied Biosystems, Foster City, CA, USA). The target genes included tight junction and mucosal barrier-related markers: *TJP1*, *TJP2*, *OCLN*, *CLDN2*, and *MUC2* as well as proinflammatory cytokines: *IL-6*, *IL-1β*, and *TNF-α*. Primer sequences used for amplification are detailed in Table 2. Actin beta served as the reference gene due to its stable expression (Ct variation < 5%). The 2^−ΔΔCt^ method was applied to quantify relative gene expression.

**Table 2:** Primer sequences for real-time quantitative PCR

Gene name^a^	Sequence (5′→3′)^b^	NCBI number
*TJP1*	F: GGCAAGTTGAAGATGGTGGT	XM_015278981.2
	R: ATGCCAGCGACTGAATTTCT	
*TJP2*	F: AGCAGACCCTGCTCAACATT	NM_204918.1
	R: GGGGAGAACGATCTGTTTGA	
*OCLN*	F: GCTGAGATGGACAGCATCAA	NM_205128.1
	R: TGCCACATCCTGGTATTGAG	
*CLDN2*	F: TCAGTGCGACATCTACAGCT	NM_001277622.1
	R: TTGAAGACGGTGCACCTCAT	
*MUC2*	F: ACCAAGCAGAAAAGCTGGAA	NM_001318434.1
	R: AAATGGGCCCTCTGAGTTTT	
*IL-6*	F: CAGGACGAGATGTGCAAGAA	NM_204628.1
	R: GAGAGCTTCGTCAGGCATTT	
*IL-1β*	F: CTGCCTGCAGAAGAAGCCT	NM_204524.2
	R: CTCCGCAGCAGTTTGGTCAT	
*TNF-α*	F: GCCCTTCCTGTAACCAGATG	NM_204267.2
	R: ACACGACAGCCAAGTCAACG	
*ACTB*	F: ATCCGGACCCTCCATTGTC	NM_205518.1
	R: AGCCATGCCAATCTCGTCTT	

^a^*TJP1* Tight junction protein 1, *TJP2* Tight junction protein 2, *OCLN* Occludin, *CLDN2* Claudin-2, *MUC2 *Mucin-2, *IL* Interleukin, *TNF-α* Tumor necrosis factor alpha, *ACTB* Actin beta

^b^*F* Forward primer, *R* Reverse primer

### Gut microbiota profiling analysis

Cecal content samples (*n* = 6 per group) were obtained under sterile conditions, immediately preserved in liquid nitrogen and subsequently stored at −80 °C for later analysis. Although each treatment group contained eight replicates, six cecal content samples with sufficient material and good visual quality were selected from each group for microbiota analysis to ensure reliable DNA extraction and sequencing. The selected samples were matched with the corresponding intestinal antioxidant, barrier function, and productive performance measurements for subsequent correlation analysis. Microbial genomic DNA was extracted from approximately 200 mg of frozen cecal digesta using a commercial DNA extraction kit (Qiagen, USA) according to the manufacturer’s instructions. DNA concentration and purity were determined using a NanoDrop 2000 spectrophotometer (Thermo Fisher Scientific, Waltham, MA, USA), and DNA integrity was verified by agarose gel electrophoresis. The V3–V4 hypervariable regions of the bacterial 16S rRNA gene were amplified using universal primers with barcode tags. PCR products were purified using magnetic beads, quantified using a Qubit 2.0 fluorometer, and used for library construction. High-throughput paired-end sequencing was performed on an Illumina platform by Novogene Bioinformatics Technology Co., Ltd. (Beijing, China). Raw paired-end reads were assigned to samples according to their unique barcodes, and barcode and primer sequences were removed. Paired-end reads were merged using FLASH software (version 1.2.11). Quality filtering of raw tags was performed using fastp software (version 0.23.1) to obtain high-quality clean tags. Chimeric sequences were identified by comparison with the SILVA database and removed using vsearch software (version 2.16.0), resulting in effective tags for downstream analysis. Operational taxonomic units (OTUs) were clustered at 97% sequence similarity using UPARSE. Representative sequences of each OTU were taxonomically assigned using the SILVA reference database. The OTU abundance table was normalized according to the sample with the lowest sequencing depth before subsequent diversity analyses. Alpha diversity indices, including Observed species, Shannon, Simpson, Chao1, and ACE, were calculated to evaluate microbial richness and diversity. Beta diversity was assessed based on weighted and unweighted UniFrac distance matrices and visualized by principal coordinate analysis (PCoA). Linear discriminant analysis effect size (LEfSe) was used to identify taxa with significantly different abundances among groups, with an LDA score threshold > 3. Spearman’s correlation analysis was performed to evaluate the relationships between cecal microbial taxa and intestinal antioxidant status, barrier function, and selected productive traits.

### Statistical analysis

Statistical analyses were performed using two-way ANOVA with the general linear model (GLM) procedure in SAS software (version 9.2; SAS Institute Inc., Cary, NC, USA), complemented by GraphPad Prism 10.0 (GraphPad Software LLC., San Diego, CA, USA) for graph generation. The statistical model used was as follows:

$$Y_{ijk} = \mu + A_{i} + T_{j} + (A \times T)_{ij} + \varepsilon_{ijk}$$

where *Y*_*ijk*_ represents the observed value of the dependent variable for the *k*-th replicate under the *i*-th level of hen age and the *j*-th level of TB supplementation; *μ* is the overall mean; *A*_*i*_ is the fixed effect of hen age; *T*_*j*_ is the fixed effect of TB supplementation; (*A* × *T*)_*ij*_ represents the interaction effect between hen age and TB supplementation; and *ε*_*ijk*_ is the random error term, which was assumed to be normally distributed with a mean of zero and constant variance.

Before analysis, the normality of residuals and homogeneity of variance were assessed using the Shapiro–Wilk test and Levene’s test, respectively. The replicate cage was considered the experimental unit for productive performance data, whereas individual hens were considered the experimental unit for egg quality, intestinal morphology, antioxidant indices, gene expression, and microbiota-related analyses. A two-way ANOVA based on least squares means was performed to evaluate the effects of hen age, TB supplementation, and their interaction. When a significant interaction effect was detected (*P* < 0.05), multiple comparisons among the four treatment groups were conducted using Tukey–Kramer’s HSD test. Differences were considered significant at *P* < 0.05 and highly significant at *P* < 0.01, whereas 0.05 ≤ *P* < 0.10 was considered a tendency. For Fig. 9, raw *P*-values from Spearman’s correlation analysis were presented and were not adjusted for multiple comparisons. Therefore, the correlation analysis was considered exploratory, and nominally significant correlations were interpreted with caution.

## Results

### Laying performance

**Fig. 1:**
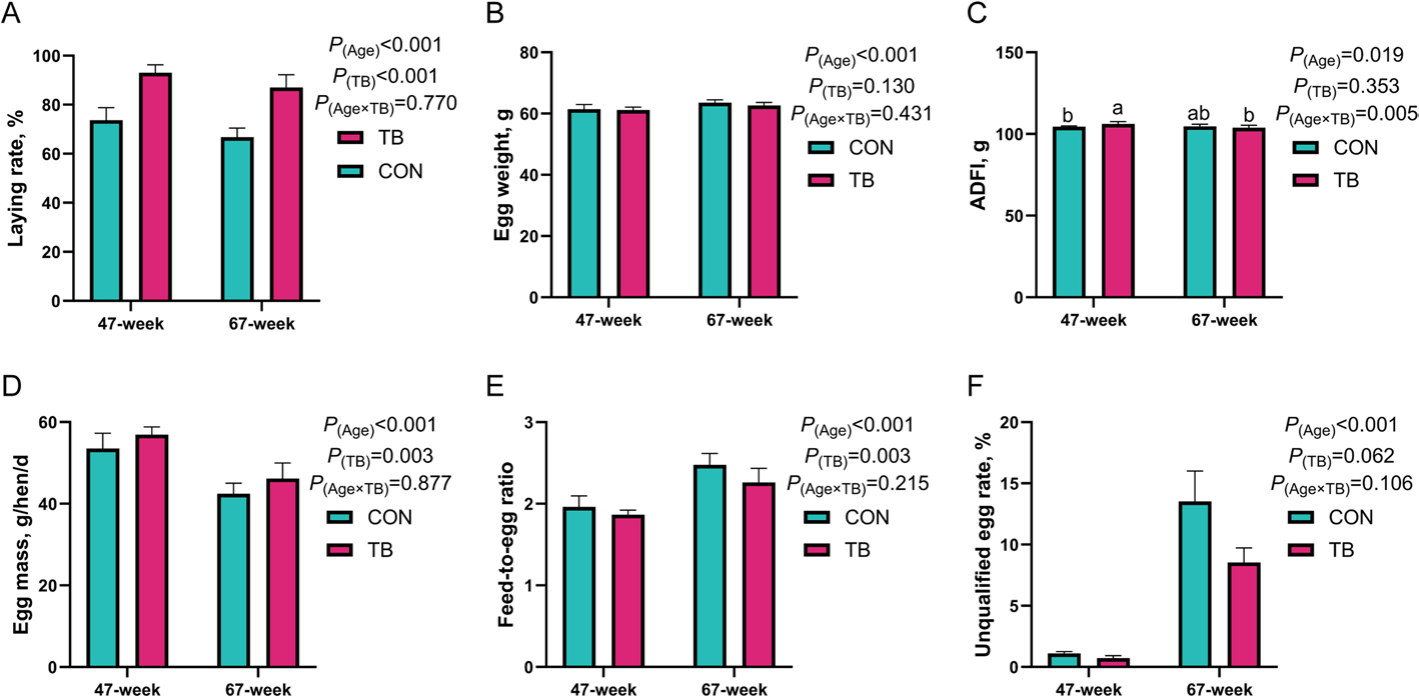
Effect of dietary theabrownin supplementation on production performance in hens of different ages. **A** Laying rate. **B** Egg weight. **C** Average daily feed intake (ADFI). **D** Egg mass. **E** Feed-to-egg ratio. **F** Unqualified egg rate. Abbreviations: CON, control group; TB, theabrownin-supplemented group; ADFI, average daily feed intake. Data are presented as mean ± SEM (*n* = 8). ^a,b^Means with different superscript within a bar differ significantly (*P* < 0.05)

As shown in Fig. 1, a significant age × TB interaction was observed for ADFI (*P* = 0.005), with TB supplementation significantly increased in 47-week-old hens. Among the parameters without a significant interaction effect, 67-week-old hens exhibited a significantly higher egg weight (*P* < 0.001), feed-to-egg ratio (*P* < 0.001), and unqualified egg rate (*P* < 0.001), but a significantly lower laying rate (*P* < 0.001) and egg mass (*P* < 0.001) than 47-week-old hens. In addition, TB supplementation significantly increased laying rate (*P* < 0.001) and egg mass (*P* = 0.003), whereas decreased feed-to-egg ratio (*P* = 0.003). Unqualified egg rate tended to be reduced by TB supplementation (*P* = 0.062). To further visualize the temporal patterns of productive performance, weekly dynamic changes in laying rate, egg weight, egg mass, feed-to-egg ratio, ADFI, and unqualified egg rate during the 12-week experimental period are shown in Supplementary Fig. S1.

### Egg quality

**Fig. 2:**
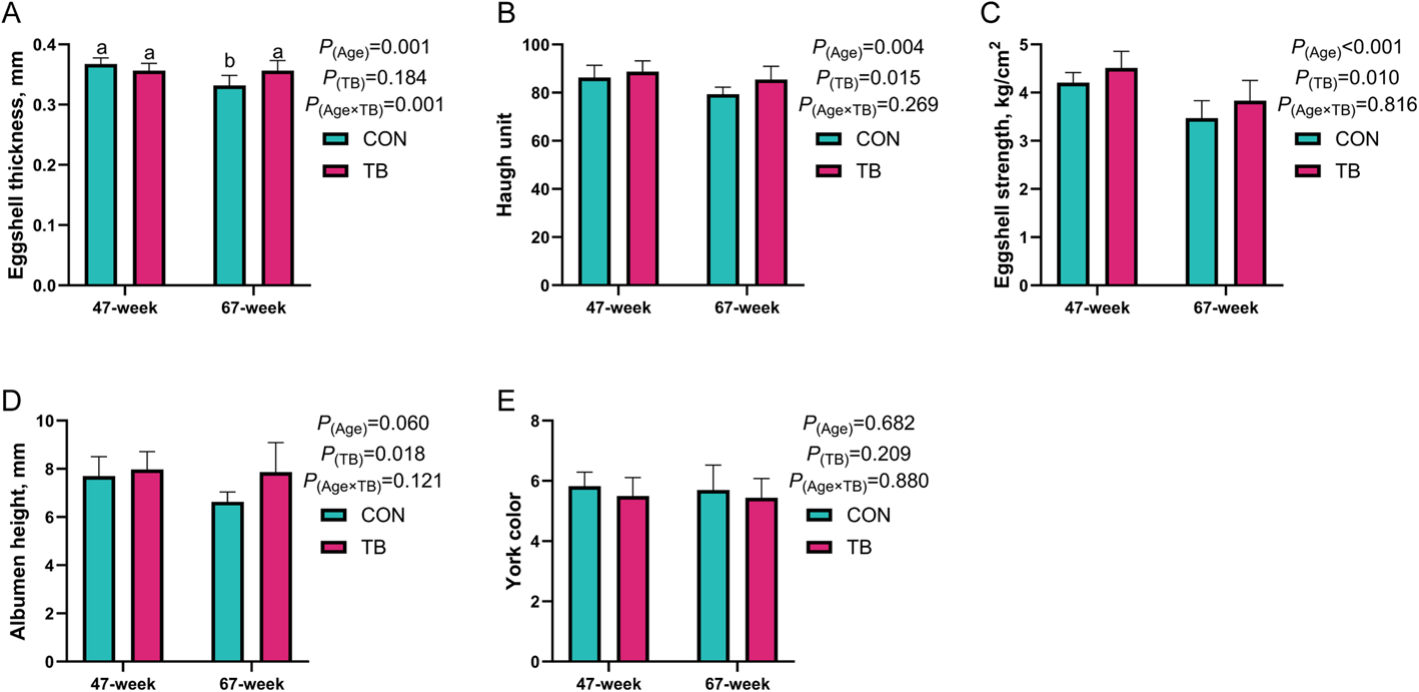
Effect of dietary theabrownin supplementation on egg quality in hens of different ages. **A** eggshell thickness. **B** Haugh unit. **C** Eggshell strength. **D** Albumen height. **E** Yolk color. Abbreviations: CON, control group; TB, theabrownin-supplemented group. Data are presented as mean ± SEM (*n* = 8). ^a,b^Means with different superscript within a bar differ significantly (*P* < 0.05)

As shown in Fig. 2, a significant age × TB interaction was observed for eggshell thickness (*P* = 0.001). 67-week-old hens showed significantly lower eggshell thickness than other groups. Among the parameters without a significant interaction effect, 67-week-old hens showed significantly lower Haugh unit (*P* = 0.004) and eggshell strength (*P* < 0.001) than 47-week-old hens. In addition, TB supplementation significantly increased Haugh unit (*P* = 0.015), eggshell strength (*P* = 0.010), and albumen height (*P* = 0.018).

### Intestinal morphology

**Fig. 3:**
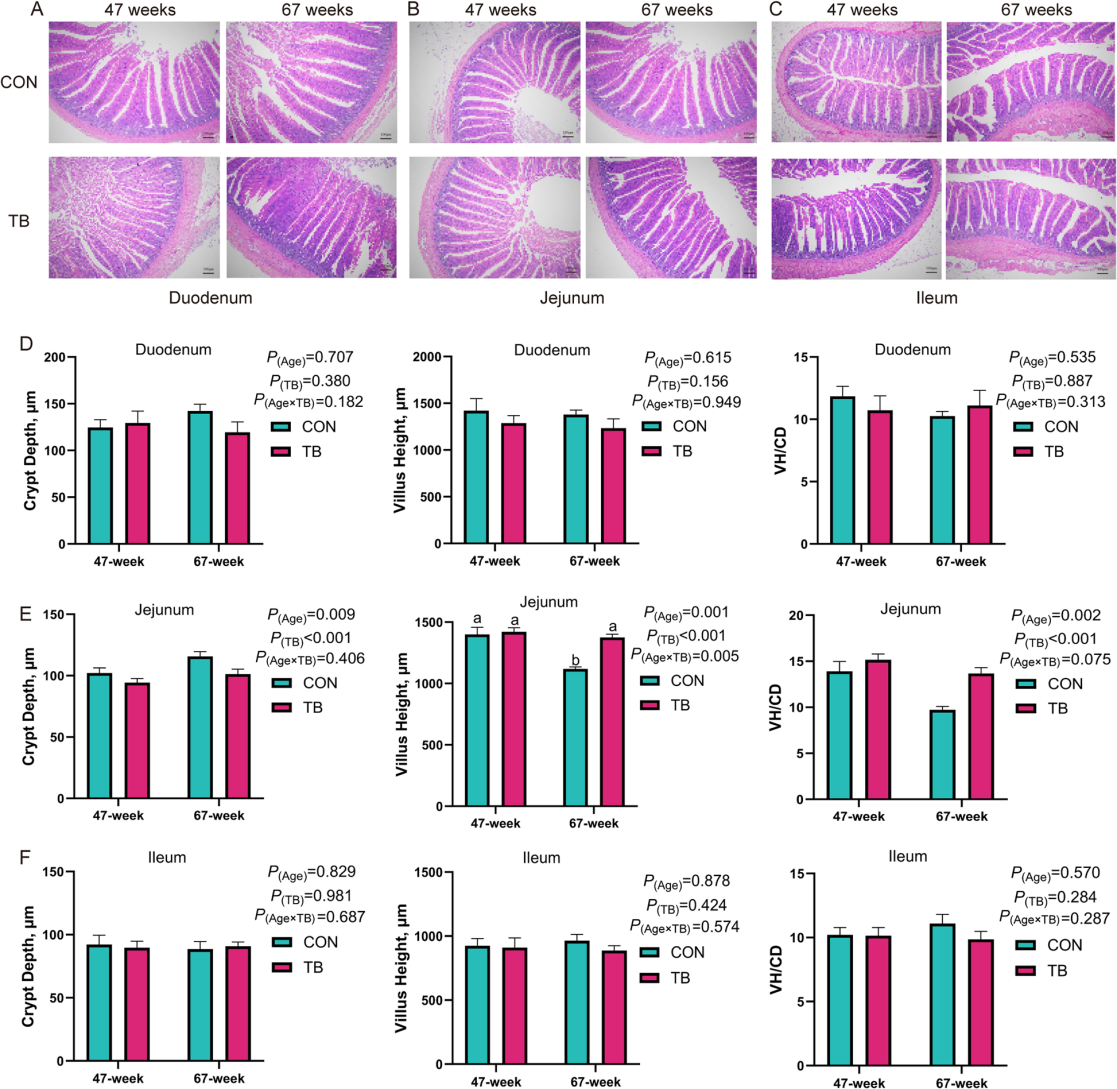
Effect of dietary theabrownin supplementation on intestinal morphology of different ages. Representative hematoxylin and eosin (H&E)-stained sections of the duodenum (**A**), jejunum (**B**), and ileum (**C**) are shown at 40 × magnification. Morphometric analysis of the duodenum (**D**), jejunum (**E**), and ileum (**F**) included crypt depth, villus height, and the VH/CD. Abbreviations: CON, control group; TB, theabrownin-supplemented group; VH/CD, villus height-to-crypt depth ratio. Data are presented as mean ± SEM (*n* = 8). ^a,b^Means with different superscript within a bar differ significantly (*P* < 0.05)

As indicated in Fig. 3, age and TB supplementation jointly exerted a significant impact on villus height (*P* = 0.005), along with a trend toward significance for the jejunum villus-to-crypt structural ratio (*P* = 0.075). At 67 weeks of age, TB supplementation significantly increased jejunal villus height compared to the control group (*P* < 0.05), whereas no significant effect was detected in 47-week-old hens. Regardless of treatment, jejunal crypt depth was significantly affected by age (*P* = 0.009), with older hens exhibiting deeper crypts than younger hens. In addition, TB supplementation also significantly decreased crypt depth (*P* = 0.015). Neither age nor TB treatment altered the intestinal architecture in the duodenal and ileal regions.

### Jejunum antioxidative capacity

**Fig. 4:**
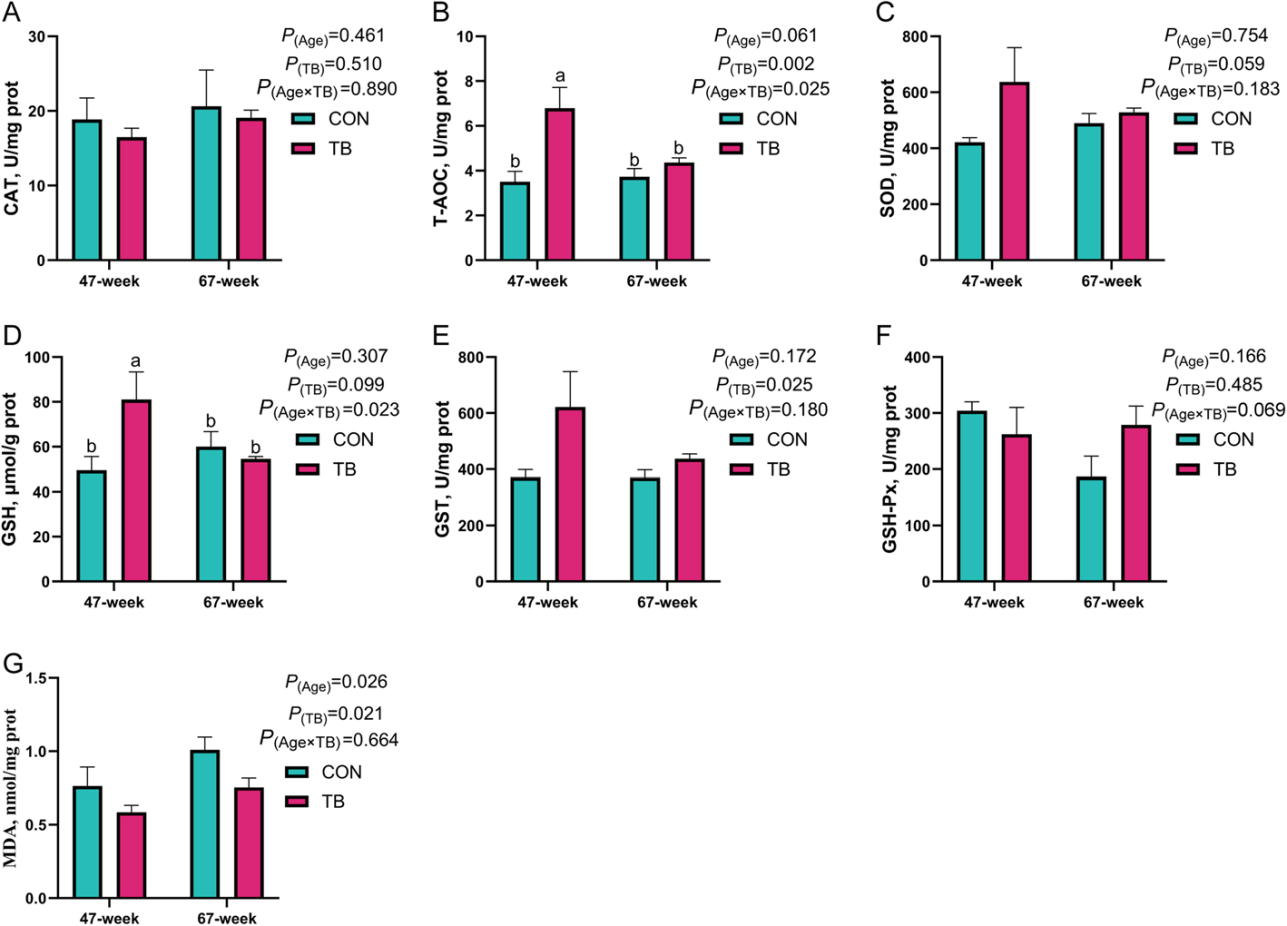
Effect of dietary theabrownin supplementation on antioxidant indices in the intestinal tissue of hens of different ages. **A** CAT. **B** T-AOC. **C** SOD. **D** GSH. **E** GST. **F** GSH-Px. **G** MDA. Abbreviations: CON, control group; TB, theabrownin-supplemented group; CAT, catalase; T-AOC, total antioxidant capacity; SOD, superoxide dismutase; GSH, glutathione; GST, glutathione S-transferase; GSH-Px, glutathione peroxidase; MDA, malondialdehyde. Data are presented as mean ± SEM (*n* = 8). ^a,b^Means with different superscript within a bar differ significantly (*P* < 0.05)

According to Fig. 4, TB supplementation led to significant increases in T-AOC (interaction, *P* = 0.025) and GSH (interaction,* P* = 0.023) concentrations, with this effect being more pronounced in 47-week-old hens than in 67-week-old hens (*P* < 0.05). TB treatment also markedly enhanced GST activity (*P* = 0.025), and this effect was independent of age. Aging increased MDA concentrations (*P* = 0.026), whereas TB supplementation mitigated this response (*P* = 0.021). In addition, a trend toward elevated SOD activity was observed with TB supplementation (*P* = 0.059).

### Jejunal tight junction- and inflammation-related gene expression

**Fig. 5:**
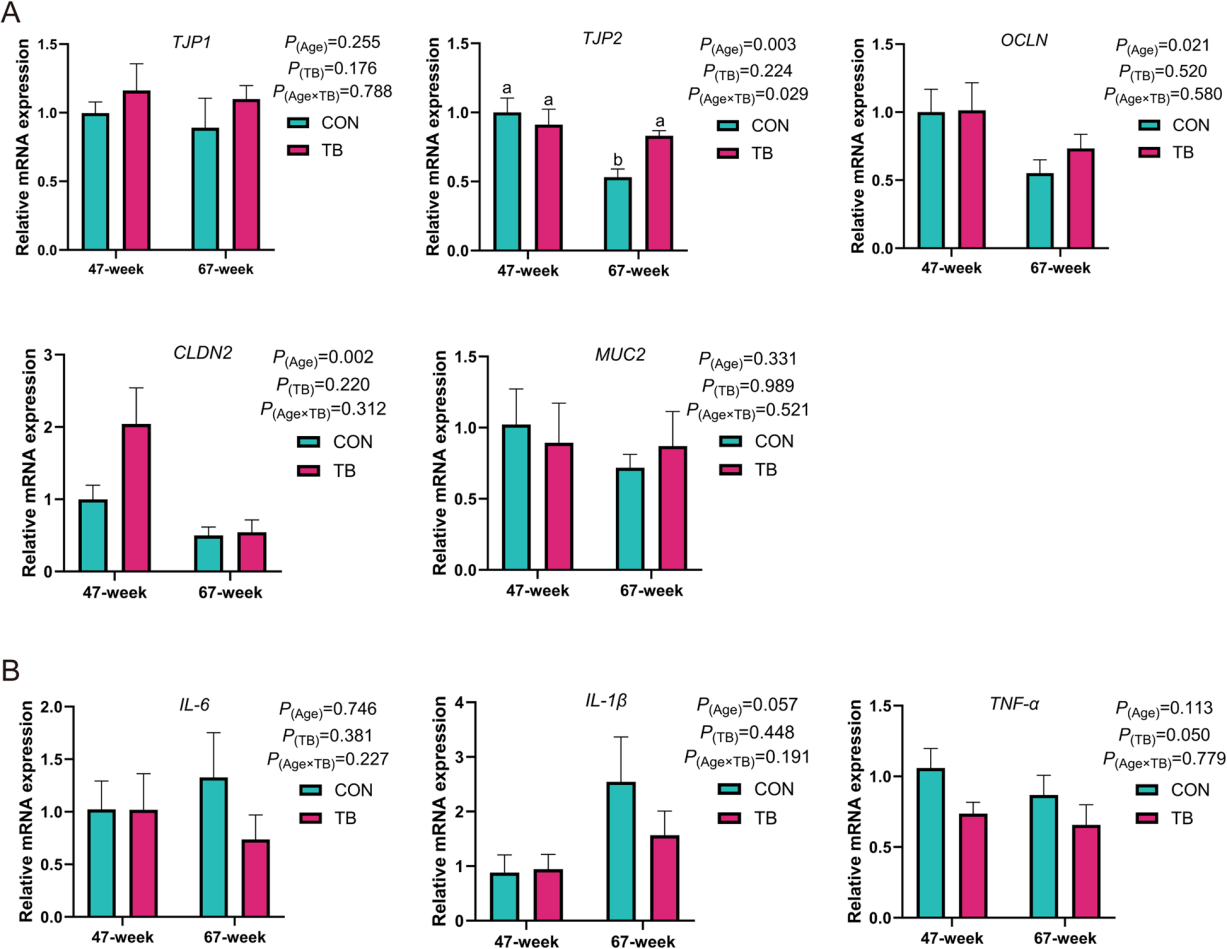
Effect of dietary theabrownin supplementation on jejunal mRNA expression of genes related to tight junction integrity and inflammatory response in hens of different ages. **A** Relative expression levels of *TJP1*, *TJP2*, *OCLN*, *CLDN2*, and *MUC2*. **B** Relative expression levels of cytokine genes *IL-6*, *IL-1β*, and *TNF-α*. Abbreviations: *TJP1*, tight junction protein 1; *TJP2*, tight junction protein 2; *OCLN*, Occludin; *CLDN2*, Claudin-2; *MUC2*, Mucin-2; *IL-6*, Interleukin-6; *IL-1β*, Interleukin-1 beta; *TNF-α*, Tumor necrosis factor-alpha. Data are presented as mean ± SEM (*n* = 8). ^a,b^Means with different superscript within a bar differ significantly (*P* < 0.05)

As shown in Fig. 5A, a significant interaction between age and TB supplementation was observed for *TJP2* expression (interaction, *P* = 0.029). At 67 weeks of age, *TJP2* mRNA levels were significantly lower than at 47 weeks in the CON group (*P* < 0.05), and TB supplementation notably increased the expression of *TJP2* in the 67-week-old laying hens (*P* < 0.05). Aging also significantly downregulated *OCLN* (*P* = 0.021) and *CLDN2* (*P* = 0.002) expression, independent of TB supplementation.

For inflammatory cytokines (Fig. 5B), aging tended to increase *IL-1β* expression (*P* = 0.057), while TB supplementation showed a marginal reduction in *TNF-α* expression (*P* = 0.050).

### Cecum microbiota diversity

**Fig. 6:**
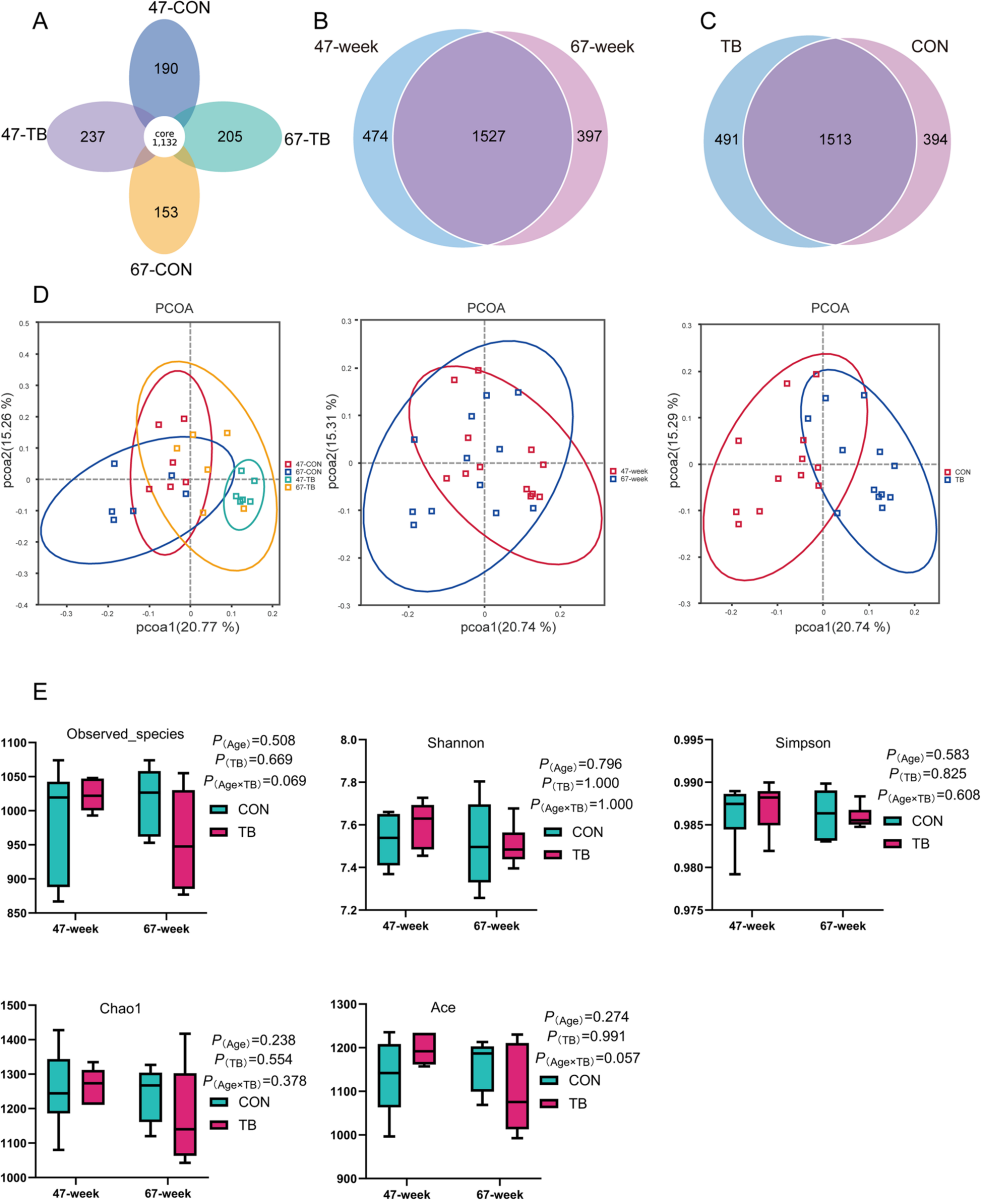
Effect of dietary theabrownin supplementation on cecal microbiota diversity in hens of different ages. **A** Venn diagram showing the number of shared and unique operational taxonomic units (OTUs) among the four groups. **B** Venn diagram showing shared and unique OTUs between 47-week and 67-week hens. **C** Venn diagram showing shared and unique OTUs between CON and TB groups. **D** Principal coordinate analysis (PCoA) based on Bray–Curtis distances, illustrating β-diversity of cecal microbiota among the four groups, age groups, and dietary groups. **E** The α-diversity indices including observed species, Shannon, Simpson, Chao1, and ACE across the four groups. Data are presented as mean ± SEM (*n* = 6)

As shown in Fig. 6, analysis revealed that 1,132 OTUs were present in all experimental groups, accompanied by 190, 237, 153, and 205 unique OTUs identified in the 47-CON, 47-TB, 67-CON, and 67-TB groups, respectively (Fig. 6A). Between age groups, 1,527 OTUs were common, while 474 and 397 were unique to the 47-week and 67-week groups (Fig. 6B). TB and CON groups shared 1,513 OTUs, with 491 and 394 specifics to each group (Fig. 6C). PCoA based on unweighted_unifrac dissimilarities showed partial clustering between groups by age and TB treatment (Fig. 6D). Alpha diversity analysis showed no significant changes in Chao1, ACE, Shannon, or Simpson indices due to either TB supplementation or age (Fig. 6E, *P* > 0.05).

### Cecum microbiota composition

**Fig. 7:**
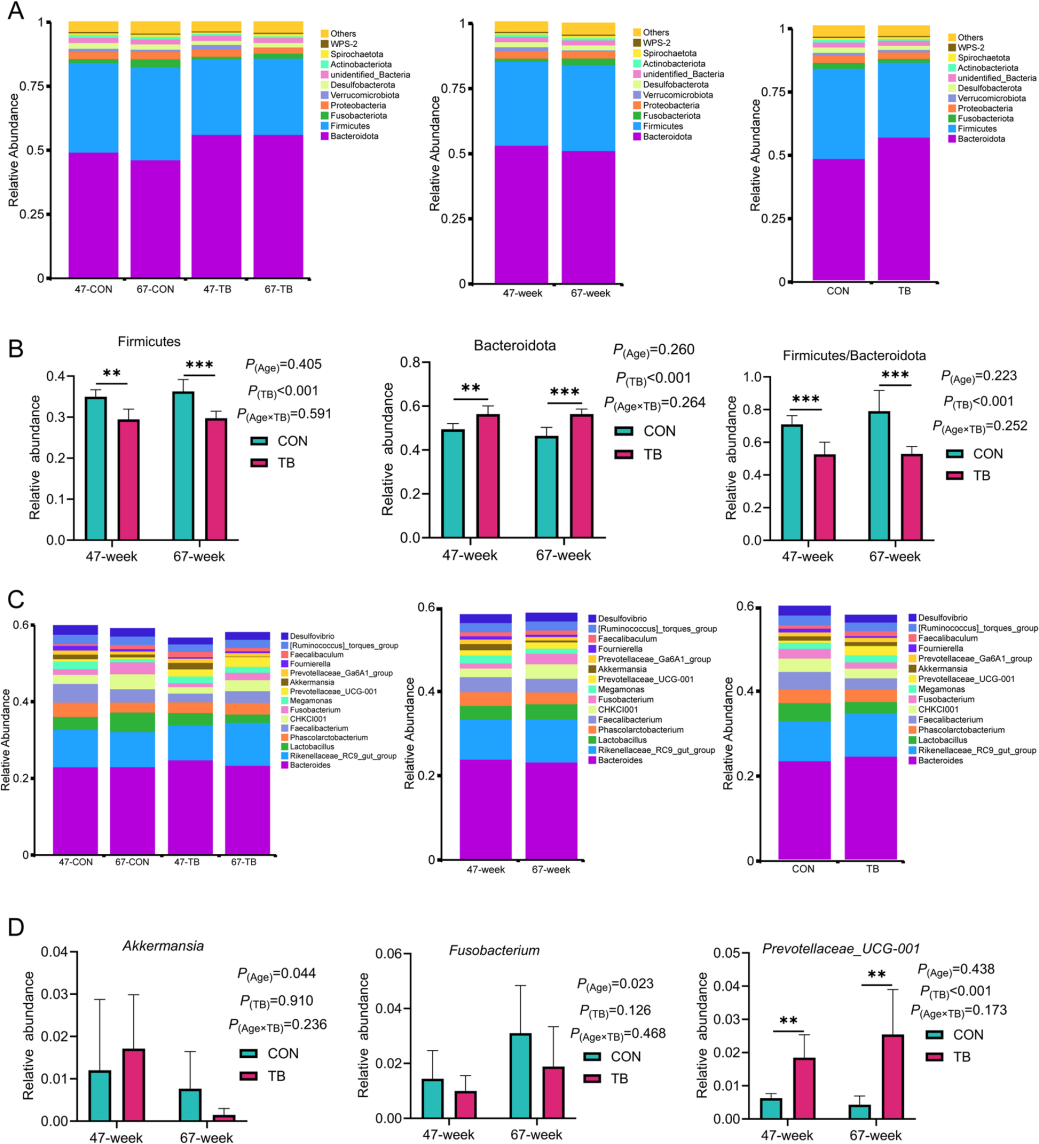
Effect of dietary theabrownin supplementation on cecum microbiota composition in hens of different ages. **A** Relative abundance of bacterial taxa at the phylum level among the four groups (47_CON, 47_TB, 67_CON, 67_TB), between age groups (47-week and 67-week), and between dietary treatments (CON and TB). **B** Comparisons of the relative abundance of major phyla (Firmicutes, Bacteroidota) and the Firmicutes/Bacteroidota ratio among groups. **C** Relative abundance of bacterial taxa at the genus level among the four groups, between age groups, and between dietary treatments. **D** Relative abundance of representative genera (*Bacteroides*, *Rikenellaceae_RC9_gut_group*, *Akkermansia*, *Fusobacterium*, and *Megamonas*) among the four groups. Data are presented as mean ± SEM (*n* = 6). ^*^P < 0.05, ^**^P < 0.01, ^***^P < 0.001

As shown in Fig. 7A, Firmicutes and Bacteroidota were the dominant bacterial phyla in the cecum of all treatment groups. At the phylum level (Fig. 7B), aging had no significant effect on the abundance of these major phyla or on the Bacteroidota to Firmicutes ratio. However, TB supplementation markedly shifted the microbial structure by increasing the proportion of Bacteroidota and reducing Firmicutes levels (*P* < 0.001), thereby leading to a significant improvement in the Bacteroidota/Firmicutes ratio. No significant interactions between age and TB supplementation were detected for any of these phylum-level taxa. At the genus level, aging was associated with a reduction in *Akkermansia* abundance (*P* = 0.044) and an elevation in *Fusobacterium* (*P* = 0.023). Additionally, TB treatment promoted the growth of *Prevotellaceae_UCG-001* (*P* < 0.001; Fig. 7C and D).

**Fig. 8:**
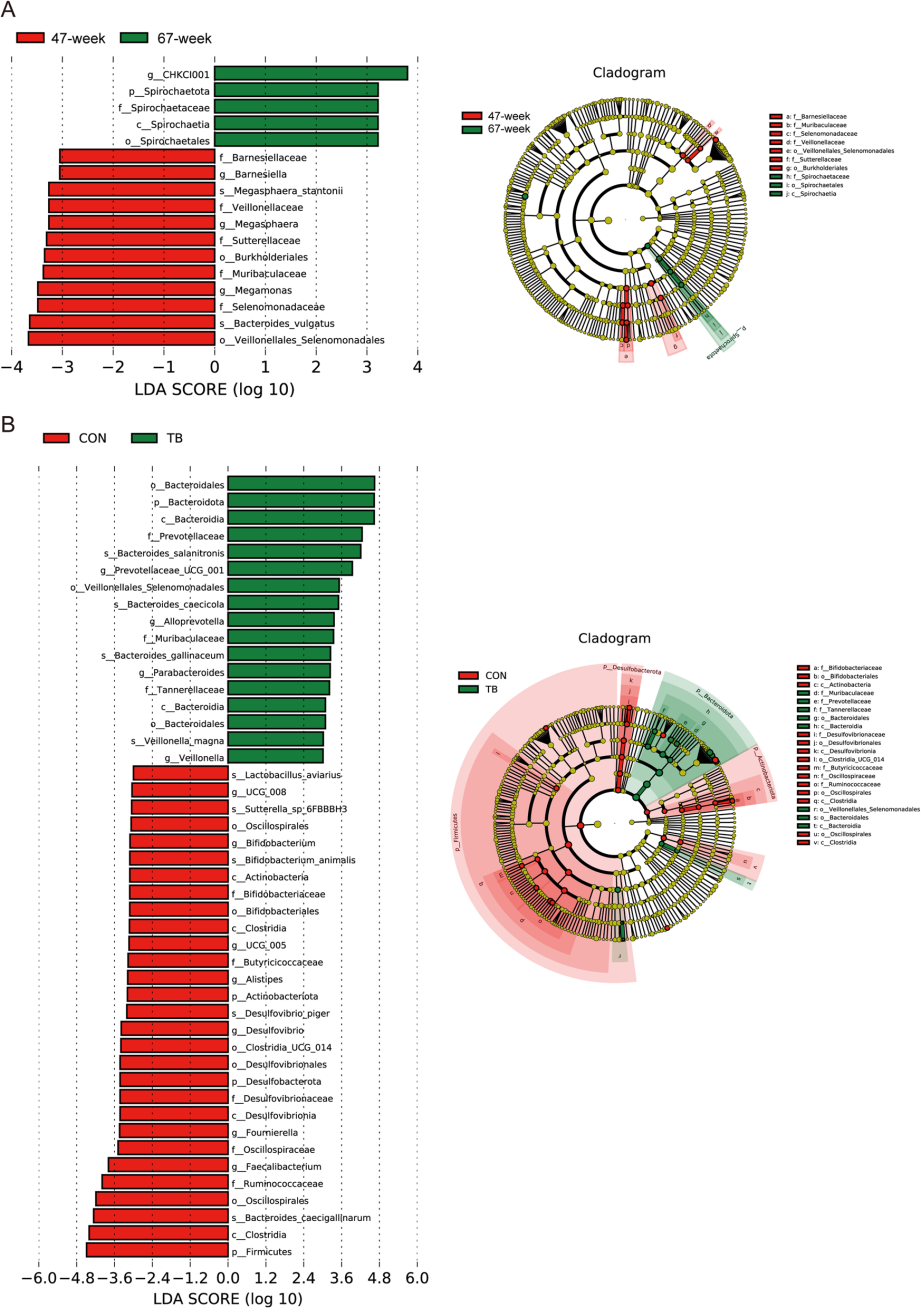
LEfSe analysis of differentially enriched cecal bacterial taxa in laying hens. **A** LDA scores and cladogram identifying bacterial taxa with significantly different abundances between age groups (47-week and 67-week) (LDA score > 3, *P* < 0.05). **B** LDA scores and cladogram showing differentially abundant taxa between dietary treatments (CON and TB) (LDA score > 3, *P* < 0.05). Red and green bars or nodes represent taxa enriched in the 47-week or CON groups and the 67-week or TB groups, respectively. Taxonomic ranks are indicated as: phylum (p), class (c), order (o), family (f), genus (g), and species (s). *n* = 6 per group

LEfSe analysis further revealed distinct microbial biomarkers between groups (LDA score > 3; Fig. 8). A total of 5 and 12 taxa were enriched in the 47-week-old and 67-week-old groups, respectively. In the 47-week-old, dominant taxa included Barnesiellaceae, *Barnesiella,* *Megasphaera*, *Megamonas*, and *Bacteroides vulgatus* (Fig. 8A). In terms of treatment effects, 17 taxa were identified as characteristic of the TB group. Notably, the TB group exhibited enrichment of Bacteroidales, Bacteroidota, Prevotellaceae, *Prevotellaceae_UCG-001*, and *Alloprevotella* (Fig. 8B).

### Correlation analysis of cecal microbiota with intestinal antioxidant status, barrier function, and production-related traits in laying hens

**Fig. 9:**
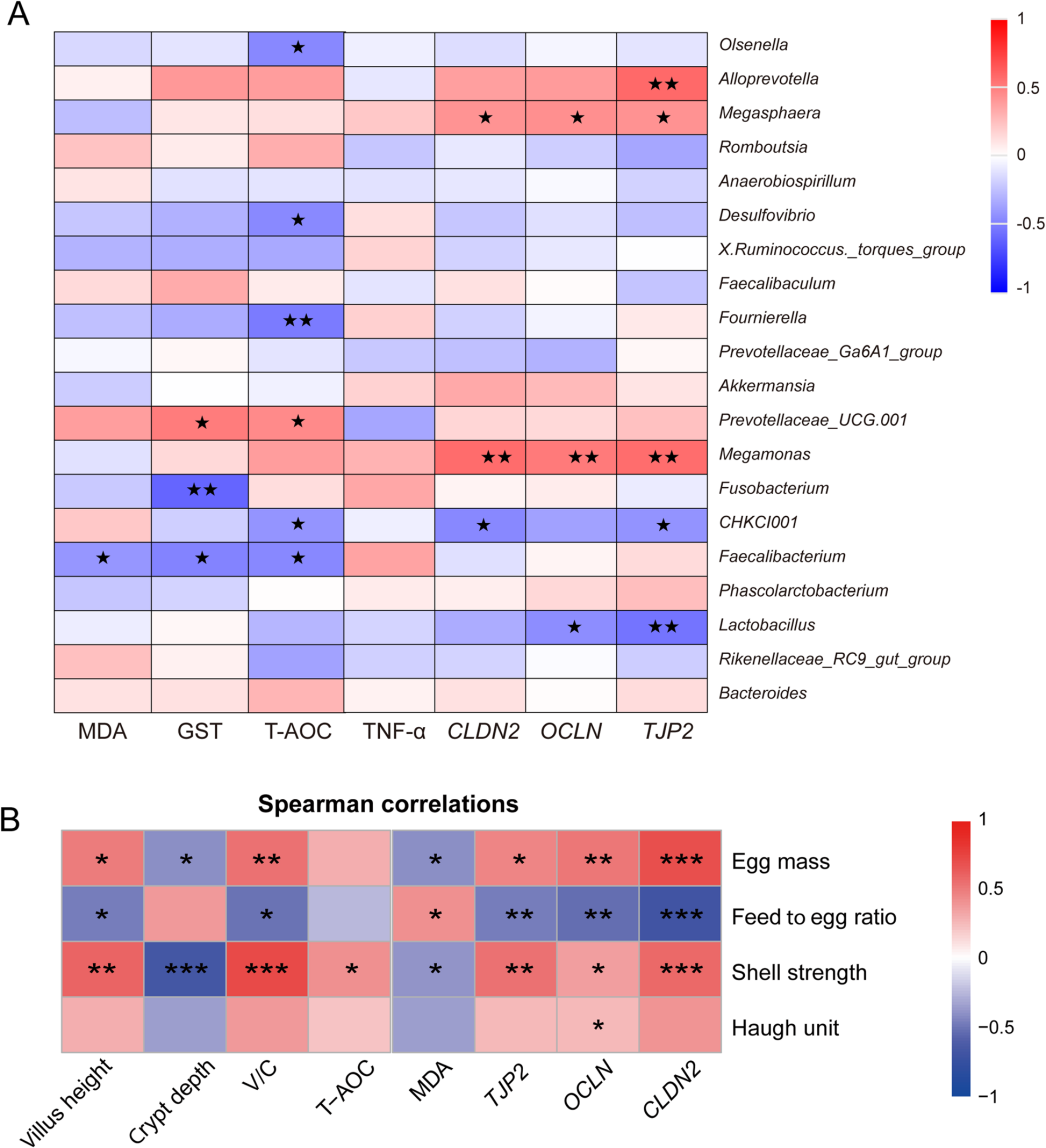
Spearman correlation analysis of cecal microbiota with intestinal antioxidant status, barrier-related gene expression, and production-related traits in laying hens. **A** Heatmap showing correlations between the relative abundances of the top 20 genera and intestinal antioxidant parameters together with barrier-related gene expression. **B** Heatmap showing correlations between representative productive and egg quality traits and intestinal structural and functional indicators. Colors represent Spearman correlation coefficients, with red indicating positive correlations and blue indicating negative correlations. Abbreviations: MDA, malondialdehyde; GST, glutathione S-transferase; T-AOC, total antioxidant capacity; TNF-α, tumor necrosis factor-α; *CLDN2*, claudin-2; *OCLN*, occludin; *TJP2*, tight junction protein 2; V/C, villus height-to-crypt depth ratio. ^*^P < 0.05, ^**^P < 0.01, ^***^P < 0.001. The *P*-values were not adjusted for multiple comparisons; therefore, these correlations should be interpreted as exploratory associations

Spearman correlation analysis revealed close associations between cecal microbial genera and intestinal antioxidant status and barrier-related gene expression in laying hens (Fig. 9A). *Prevotellaceae_UCG-001* was positively correlated with GST and T-AOC, whereas *Faecalibacterium* showed negative correlations with MDA, GST, and T-AOC. In addition, *Fusobacterium* was negatively associated with GST, and *Olsenella*, *Desulfovibrio*, *Fournierella*, and *CHKCI001* were negatively correlated with T-AOC. With respect to barrier-related genes, *Megasphaera* and *Megamonas* were positively correlated with *CLDN2*, *OCLN*, and *TJP2*, while *Alloprevotella* was positively associated with *TJP2*. In contrast, *CHKCI001* was negatively correlated with *CLDN2* and *TJP2*, and *Lactobacillus* was negatively associated with *OCLN* and *TJP2*.

Correlation analysis further showed that productive and egg quality traits were closely associated with intestinal structural and functional indicators (Fig. 9B). Egg mass and shell strength were positively correlated with villus height, V/C, *TJP2*, *OCLN*, and *CLDN2*, but negatively correlated with crypt depth and MDA. In contrast, the feed-to-egg ratio showed the opposite pattern. Haugh unit was positively correlated with *OCLN*.

## Discussion

Advancing age in laying hens is often accompanied by reduced productive efficiency and deterioration in egg quality, which is closely related to impaired nutrient digestion and utilization rather than chronological age alone [29]. In the present study, older hens produced heavier eggs but showed poorer feed efficiency, lower egg mass, and a higher proportion of unqualified eggs, indicating that increased egg size in late-laying hens does not necessarily reflect improved productive status. Rather, this pattern suggests that aging shifts nutrient partitioning away from efficient production and toward maintenance demands, so that more feed is required to sustain egg output while the proportion of qualified eggs declines. This interpretation is consistent with previous evidence showing that late-phase hens often maintain or increase egg weight despite a progressive decline in production efficiency and egg quality [30, 31]. The deterioration in egg quality observed in older hens further supports this view. Reduced eggshell thickness and eggshell strength in aged hens are commonly linked to a lower capacity for calcium absorption, transport, and deposition, which becomes increasingly limiting as egg size increases during the late laying period [32]. Under these conditions, the demand for shell formation may exceed the physiological capacity of hens to maintain normal shell mineralization, resulting in larger but structurally weaker eggs. In addition, the decline in albumen quality may reflect impaired magnum secretory activity and reduced antioxidant-metabolic support for normal egg formation in older hens [33]. Therefore, the age-related changes observed here likely reflect a broader decline in the efficiency and stability of the biological processes required for producing high-quality eggs, rather than changes in a single trait alone. Against this background, the beneficial effects of TB are physiologically meaningful because they were accompanied not only by improved productive performance and egg quality, but also by better intestinal status. Dietary TB supplementation improved feed efficiency, egg mass, and several egg quality traits, suggesting that TB may have supported the physiological conditions required for efficient nutrient utilization and egg formation [34]. This improvement may be partly related to the intestinal effects of TB observed in the present study. Improved intestinal morphology and barrier function can help maintain the absorptive surface and epithelial integrity, thereby supporting nutrient and mineral utilization. Because eggshell mineralization depends heavily on calcium absorption and transport, intestinal improvements may indirectly contribute to better shell quality by facilitating calcium utilization. Calcium transport-related proteins, such as TRPV6, calbindin-D28k, and PMCA1b, have been reported to participate in calcium transport and eggshell formation [35]. In addition, TB-induced modulation of the cecal microbiota may favor microbial fermentation and SCFA-related metabolic activity. SCFAs can support epithelial energy metabolism and barrier function and may also enhance mineral solubility and absorption partly through regulation of luminal pH [36]. Importantly, the greater responsiveness observed in older hens suggests that the effects of TB may depend on the physiological status of the birds and may therefore be more pronounced during the late laying period. This interpretation is biologically plausible, as older hens generally exhibit impaired digestive function, reduced nutrient retention, and greater oxidative and reproductive challenges, and may therefore respond more strongly to dietary interventions that help maintain nutrient metabolism and tissue function [37]. Given the known antioxidant and gut-supportive activities of tea-derived polyphenols, TB may have alleviated age-related physiological stress and thereby improved the internal conditions required for efficient egg production [23], although this mechanism still requires further verification. Thus, rather than simply stimulating output, TB may have improved the physiological basis required for sustained production in aged laying hens.

The morphology of intestinal villi and crypts plays a crucial role in determining absorptive efficiency, and alterations such as reduced villus height or increased crypt depth can markedly compromise nutrient uptake [38]. In the present study, we observed significant age-related alterations in jejunal morphology, including reduced villus height, increased crypt depth, and a decreased V/C ratio in 67-week-old hens. These findings are consistent with previous reports of mucosal aging in poultry [39] and suggest that intestinal aging may reduce the effective absorptive surface while increasing epithelial renewal demand. Increased crypt depth in older hens may reflect compensatory epithelial turnover in response to mucosal stress or low-grade inflammation, potentially leading to greater maintenance energy demands and reduced efficiency of nutrient utilization for egg production [40]. Dietary inclusion of 100 mg/kg TB significantly improved jejunum morphology, particularly by increasing villus height and the V/C ratio. This intestinal segment-specific response is consistent with the findings of Zhang et al. [41], who reported that TB supplementation enhanced villus height primarily in the duodenum and jejunum, with minimal changes observed in the ileum. Interestingly, the morphological improvements in the jejunum were more pronounced in 67-week-old hens, suggesting a possible interaction between intestinal region and host age in responsiveness to TB intervention. One possible explanation is that aging intestines, particularly in functionally active regions like the jejunum, may experience greater oxidative burden and structural decline, making them more sensitive to antioxidant modulation [42]. Because the jejunum is a major site of nutrient absorption, this structural improvement may be directly relevant to the better feed efficiency, egg mass, and egg quality observed in TB-supplemented hens. Our findings align with previous studies in mammalian and avian models showing that tea-derived polyphenols promote epithelial renewal and intestinal barrier integrity [43]. Tea polyphenols have also been reported to improve intestinal barrier function and exert antioxidant and anti-inflammatory effects in livestock, supporting the possibility that TB preserved jejunal morphology partly through improved redox and epithelial homeostasis [44]. This interpretation is further supported by the correlation analysis, in which egg mass, feed efficiency, and egg quality traits were significantly associated with intestinal structural and functional indicators, suggesting that the intestinal benefits of TB may be functionally linked to its productive responses.

Oxidative stress plays a pivotal role in intestinal aging, as the gastrointestinal epithelium is constantly exposed to reactive oxygen species from both endogenous metabolism and dietary factors [45–47]. Elevated oxidative damage, as indicated by reduced antioxidant enzyme activity, impairs cellular structures, disrupts mucosal defense, and accelerates inflammatory signaling [48, 49]. Consistent with this, the current research found that aging significantly elevated MDA levels in the jejunum, reflecting enhanced lipid peroxidation and oxidative damage. Meanwhile, dietary supplementation with TB effectively mitigated oxidative stress by increasing T-AOC and GST activity. Similar effects have been reported in earlier studies in mammalian models where tea-derived polyphenols enhanced cellular redox balance via upregulation of endogenous antioxidant systems [50]. Interestingly, the antioxidant enhancement of T-AOC was more pronounced in younger hens (47 weeks), suggesting an age-dependent responsiveness. This may be due to reduced bioavailability or metabolic responsiveness of the aging gut, as older animals often exhibit lower polyphenol absorption efficiency and Nrf2 pathway sensitivity [51]. Alternatively, the higher baseline oxidative burden in older hens may have exceeded the compensatory capacity of a moderate TB dose, thereby limiting its detectable effects on antioxidant parameters. This may suggest a dose-dependent antioxidant response to TB, with higher supplementation levels, such as 400 mg/kg, potentially being required to elicit more pronounced antioxidant effects in aged laying hens [28]. Nevertheless, in the present study, 100 mg/kg TB was selected as a practical supplementation level and was sufficient to improve intestinal health and productive performance, indicating that this dose may be appropriate when the primary objective is to alleviate age-related declines in production through intestinal health improvement rather than to maximize antioxidant capacity.

The intestinal epithelial barrier relies on tight junction proteins such as ZO, OCLN, and CLDN to regulate paracellular permeability. Age-associated declines in these proteins compromise barrier integrity, increasing susceptibility to microbial translocation and systemic inflammation [52, 53]. Studies in rodent models have revealed that gut aging is accompanied by epithelial degeneration, including villus shortening and a decline in the expression of tight junction proteins such as OCLN and ZO-1 [54]. In the present study, *TJP2* mRNA expression declined significantly with age but was restored by TB supplementation in 67-week-old birds, indicating that TB may exert barrier-protective effects under age-compromised conditions. Although aging also downregulated *OCLN* and *CLDN2* expression independent of treatment, the recovery of *TJP2* in older hens suggests that TB may still confer partial protection to the epithelial barrier under compromised conditions. It is well established that inflammatory cytokines can negatively regulate tight junction protein expression. Among them, TNF-α predominantly produced by activated T lymphocytes and macrophages, is considered a factor in the reduction of *OCLN* levels [53, 55]. Previous research in mice indicated that elevated TNF-α impairs intestinal barrier integrity by downregulating *Ocln* expression in the epithelial layer [56]. In the current study, TB tended to reduce TNF-α and IL-1β expression, suggesting an anti-inflammatory role that may indirectly contribute to tight junction preservation in aging hens. Previous studies have demonstrated that TNF-α and IL-1β can disrupt tight junction protein expression and promote epithelial permeability by activating NF-κB and MAPK pathways [57, 58]. Polyphenols, including TB, have been shown to inhibit these pro-inflammatory pathways, thereby reinforcing epithelial barrier function [59]. Taken together, these results suggest that the beneficial effects of TB on intestinal health may involve coordinated regulation of redox balance, inflammatory tone, and barrier maintenance rather than a single pathway alone.

Gut microbial communities contribute significantly to host metabolism, immune function, and epithelial barrier maintenance. A balanced microbial community supports nutrient extraction and synthesis, modulates mucosal and systemic immune responses, and reinforces epithelial tight junctions, thereby sustaining intestinal homeostasis [60, 61]. In the present study, neither age nor TB supplementation significantly altered alpha diversity indices, suggesting that overall microbial richness and evenness were preserved across treatments. In poultry, the cecal microbiota is dominated by Firmicutes and Bacteroidota. Variations in the Firmicutes/Bacteroidota (F/B) ratio have been associated with differences in energy harvest efficiency and metabolic health [62, 63]. Although many nutritional interventions such as high-energy diets or probiotics have been reported to increase the F/B ratio [63–66]. TB supplementation in the present study significantly decreased the F/B ratio by increasing the relative abundance of Bacteroidota while reducing Firmicutes. This divergence from the predominant trend may be attributed to the unique biochemical properties of TB, which are high-molecular-weight polyphenols with poor small-intestinal absorption but extensive microbial fermentation in the hindgut. A similar decrease in the F/B ratio was also observed by Lei et al. in a D-galactose-induced aging mouse model, in which TB supplementation significantly increased Bacteroidota abundance while reducing Firmicutes [22]. Polyphenols have been shown to selectively promote Bacteroidota lineages, particularly members of the Bacteroidales order, which are efficient degraders of complex polysaccharides and potential producers of SCFAs [67]. The competitive advantage conferred to Bacteroidota under TB supplementation may be related to the inhibitory effects of polyphenolic compounds on certain Firmicutes taxa, along with providing a substrate niche favorable to Bacteroidota growth. From a functional standpoint, a decreased F/B ratio may be associated with enhanced fiber fermentation capacity, reduced fat deposition, and improved gut barrier function [68]. Consistent with our findings, dietary supplementation with cottonseed meal fermented by *Candida tropicalis* increased the relative abundance of Bacteroidota in the cecum in broilers [69]. At the genus level, our study revealed the TB-induced reduction in the F/B ratio coincided with increased *Prevotellaceae_UCG-001* and *Alloprevotella* abundance, which ferment dietary polysaccharides into SCFAs such as butyrate and propionate. These metabolites are involved in cellular energy supply for colonocytes and also exhibit anti-inflammatory properties while enhancing tight junction integrity, which collectively benefits the intestinal barrier [70, 71]. Therefore, the enrichment of these taxa may provide a microbial explanation for the improved intestinal morphology, barrier-related gene expression, and productive responses observed in TB-supplemented hens. One of the most notable age-related changes was a significant reduction in *Akkermansia* abundance in older hens. *Akkermansia muciniphila* is well known for its ability to degrade mucin, stimulate mucus layer renewal, and modulate host immune responses [72, 73]. A reduction in *Akkermansia* abundance has been associated with weakened mucosal barrier function, heightened inflammation, and metabolic disturbances in both avian and mammalian systems [74]. The decline observed in older hens here may therefore reflect diminished mucosal maintenance capacity and could contribute to the age-associated reductions in tight junction expression observed in this study. On the other hand, aging was associated with increased *Fusobacterium*, a genus frequently linked to intestinal inflammation, epithelial barrier disruption, and pathogenic potential in poultry and other species [75]. The correlation analysis between cecal microbiota and jejunal antioxidant and barrier parameters in our study revealed several taxa potentially involved in modulating oxidative status in aged laying hens. Notably, *Faecalibacterium* abundance was negatively associated with MDA levels, supporting its recognized role as a butyrate-producing genus with anti-inflammatory and redox-modulating properties. This observation is consistent with recent findings that link *Faecalibacterium* enrichment to improved antioxidant enzyme activity and reduced oxidative stress in mammalian models [76, 77]. Conversely, genera such as *Fusobacterium*, *Desulfovibrio*, and *Olsenella* exhibited negative correlations with antioxidant markers, aligning with studies reporting their involvement in pro-inflammatory and pro-oxidative pathways [78]. Interestingly, *Prevotellaceae_UCG-001* showed positive associations with GST and T-AOC, which may reflect its capacity to ferment complex polysaccharides into SCFAs with known antioxidant and anti-inflammatory potential. However, previous reports on *Prevotellaceae* are inconsistent, with some studies suggesting context-dependent roles in either promoting or alleviating oxidative stress [79]. These discrepancies may stem from differences in host species, dietary composition, microbial competition, and intestinal sampling sites. In this study, *Megasphaera*, *Megamonas,* and *Alloprevotella* exhibited positive correlations with the expression of tight junction proteins such as *CLDN2*, *OCLN*, and *TJP2*, suggesting a supportive role of SCFA-producing bacteria in maintaining intestinal barrier integrity. This is consistent with previous reports that *Megasphaera elsdenii* enhances mucosal barrier function by converting lactate into butyrate, a key SCFA that fuels epithelial cells and promotes tissue renewal [80]. Likewise, *Alloprevotella* has been positively associated with antioxidant capacity in livestock models [81]. In contrast, the negative correlations of *CHKCI001* and *Lactobacillus* with tight junction expression were unexpected. *CHKCI001* may plausibly act as an opportunistic taxon that disrupts mucosal barrier function. However, the inverse association observed with *Lactobacillus*, a genus generally recognized for its probiotic properties, raises questions regarding strain-specific differences or host-dependent effects. Variability in the effect of *Lactobacillus* on tight junction maintenance has been observed across different strains. For instance, only certain strains such as *L. acidophilus *LA1 significantly upregulate tight junction protein expression through TLR2-mediated pathways, whereas others exert minimal or even adverse effects depending on the host's physiological context [82]. Similarly, in human models, *L. plantarum* has shown variable effects on the expression of genes associated with epithelial barrier function [83]. In the present study, the genus-level resolution of 16S rRNA sequencing could not distinguish specific *Lactobacillus* strains, and the observed association may also have been influenced by the altered intestinal microenvironment of aged laying hens and TB-induced shifts in the overall cecal microbial community. Therefore, the barrier-related effects of TB may depend more on the coordinated microbial community response than on the abundance of a single bacterial genus. More importantly, the present correlation analysis identified *Prevotellaceae_UCG-001* as a particularly noteworthy taxon, because it was enriched by TB supplementation and positively associated with intestinal antioxidant indices. Combined with the significant associations between productive or egg quality traits and intestinal indicators, this finding suggests that *Prevotellaceae_UCG-001* may represent a potential microbial taxon linking improved intestinal health to better productive responses in aged laying hens. Overall, these data support a model in which TB alleviates age-related productive decline partly through coordinated improvements in jejunal morphology, redox balance, barrier function, and microbial homeostasis.

The purity of the TB product used in this study was 81%, and the detailed composition of the remaining 19% was not available because only TB purity was assayed by the supplier. Previous studies have indicated that tea-derived TB preparations are complex heterogeneous mixtures or complexes containing accompanying constituents such as polysaccharides, residual polyphenols/flavonoids, proteins, lipids, caffeine, ash, and moisture [84]. Some of these non-TB constituents may possess biological activities, including antioxidant, anti-inflammatory, or microbiota-modulating effects. However, at the supplementation level of 100 mg/kg diet, the non-TB fraction accounted for approximately 19 mg/kg diet. Thus, the observed effects were considered to be mainly attributable to TB, although minor contributions from these accompanying components cannot be completely excluded.

## Conclusion

This study demonstrates that aging in laying hens is accompanied by declines in productive performance and egg quality, which may be partly associated with impaired intestinal health, as reflected by jejunal structural deterioration, weakened antioxidant capacity, reduced barrier-related gene expression, and altered cecal microbial composition. Dietary TB supplementation partly alleviated these age-related declines in productive performance and egg quality, while simultaneously improving jejunal morphology, redox balance, barrier function, and cecal microbial composition. Notably, productive and egg quality traits were closely associated with intestinal structural and functional indicators, and the TB-enriched genus *Prevotellaceae_UCG-001* was positively correlated with GST and T-AOC, suggesting that microbial modulation may be involved in the intestinal benefits of TB. Collectively, these findings suggest that the beneficial effects of TB on productive performance may be partly associated with the maintenance of intestinal health in aged laying hens.

## Supplementary Information

Additional file 1: Fig. S1. Weekly dynamic changes in productive performance during the 12-week experimental period.

## Data Availability

The raw 16S rRNA gene sequencing data generated in this study have been deposited in the NCBI Sequence Read Archive under BioProject accession number PRJNA1463479. Other relevant data supporting the findings of this study are available from the corresponding author upon reasonable request.

## Abbreviations

ADFI: Average daily feed intake
ACE: Abundance-based coverage estimator
CAT: Catalase
CLDN2: Claudin-2
GSH: Glutathione
GSH-Px: Glutathione peroxidase
GST: Glutathione S-transferase
H&E: Hematoxylin and eosin
IL-1β: Interleukin-1 beta
IL-6: Interleukin-6
LEfSe: Linear discriminant analysis effect size
MUC2: Mucin-2
OCLN: Occludin
OTU: Operational taxonomic unit
PCoA: Principal coordinate analysis
qPCR: Quantitative real-time polymerase chain reaction
SOD: Superoxide dismutase
T-AOC: Total antioxidant capacity
TB: Theabrownins
TJP1: Tight junction protein 1
TJP2: Tight junction protein 2
TNF-α: Tumor necrosis factor alpha
V/C: Villus height-to-crypt depth ratio

## References

[1] Réhault-Godbert S, Guyot N, Nys Y. The Golden Egg: Nutritional value, bioactivities, and emerging benefits for human health. Nutrients. 2019. 10.3390/nu11030684. 10.3390/nu11030684 PMC6470839 30909449

[2] Lutter C, Iannotti L, Stewart C. The potential of a simple egg to improve maternal and child nutrition. Matern Child Nutr. 2018. 10.1111/mcn.12678. 10.1111/mcn.12678 PMC6865885 30332538

[3] Liu XT, Lin X, Mi Y, Zeng W, Zhang C. Age-related changes of yolk precursor formation in the liver of laying hens. J Zhejiang Univ Sci B. 2018;19:390–9. 10.1631/jzus.b1700054. 29732750 10.1631/jzus.B1700054 PMC5962516

[4] Gao Z, Zheng J, Xu G. Molecular mechanisms and regulatory factors governing feed utilization efficiency in laying hens: insights for sustainable poultry production and breeding optimization. Int J Mol Sci. 2025;26(13):6389. 10.3390/ijms26136389. 40650167 10.3390/ijms26136389 PMC12250216

[5] Chen F, Zhang H, Du E, Jin F, Zheng C, Fan Q, et al. Effects of magnolol on egg production, egg quality, antioxidant capacity, and intestinal health of laying hens in the late phase of the laying cycle. Poult Sci. 2021;100(2):835–43. 10.1016/j.psj.2020.10.047. 33518137 10.1016/j.psj.2020.10.047 PMC7858092

[6] Li H, Gu Y, Jin R, He Q, Zhou Y. Effects of dietary rutin supplementation on the intestinal morphology, antioxidant capacity, immunity, and microbiota of aged laying hens. Antioxidants. 2022;11(9):1843. 10.3390/antiox11091843. 36139918 10.3390/antiox11091843 PMC9495371

[7] Goodarzi N, Akbari Bazm M, Poladi S, Rashidi F, Mahmoudi B, Abumandour MMA. Histology of the small intestine in the common pheasant (*Phasianus colchicus*): A scanning electron microscopy, histochemical, immunohistochemical, and stereological study. Microsc Res Tech. 2021;84(10):2388–98. 10.1002/jemt.23794. 33908129 10.1002/jemt.23794

[8] Horowitz A, Chanez-Paredes SD, Haest X, Turner JR. Paracellular permeability and tight junction regulation in gut health and disease. Nat Rev Gastroenterol Hepatol. 2023;20(7):417–32. 10.1038/s41575-023-00766-3. 37186118 10.1038/s41575-023-00766-3 PMC10127193

[9] Groschwitz KR, Hogan SP. Intestinal barrier function: molecular regulation and disease pathogenesis. J Allergy Clin Immunol. 2009;124:3–20. 10.1016/j.jaci.2009.05.038. 10.1016/j.jaci.2009.05.038 PMC4266989 19560575

[10] Capaldo CT, Powell DN, Kalman D. Layered defense: how mucus and tight junctions seal the intestinal barrier. J Mol Med (Berl). 2017;95(9):927–34. 10.1007/s00109-017-1557-x. 28707083 10.1007/s00109-017-1557-x PMC5548832

[11] Meyer F, Wendling D, Demougeot C, Prati C, Verhoeven F. Cytokines and intestinal epithelial permeability: A systematic review. Autoimmun Rev. 2023;22(6):103331. 10.1016/j.autrev.2023.103331. 37030338 10.1016/j.autrev.2023.103331

[12] Salazar N, Arboleya S, Fernández-Navarro T, De Los Reyes-Gavilán CG, Gonzalez S, Gueimonde M. Age-associated changes in gut microbiota and dietary components related with the immune system in adulthood and old age: a cross-sectional study. Nutrients. 2019. 10.3390/nu11081765. 10.3390/nu11081765 PMC6722604 31370376

[13] Zhang Y, Li Y, Fang X, Li X, Hou F, Tao Z, et al. Dietary capsaicin supplementation improves production performance and intestinal health of laying hens by regulating intestinal microbiota. Anim Nutr. 2025. 10.1016/j.aninu.2025.02.009. 40919308 10.1016/j.aninu.2025.02.009 PMC12408253

[14] Liang X, Fu Y, Niu K, Zhai Z, Shi H, Wang R, et al. Dietary *Eucommia ulmoides* leaf extract improves laying performance by altering serum metabolic profiles and gut bacteria in aged laying hens. Anim Nutr. 2023;15:307–19. 10.1016/j.aninu.2023.07.008. 38053802 10.1016/j.aninu.2023.07.008 PMC10694046

[15] Xiong S, Zhang Q, Zhang K, Wang J, Bai S, Zeng Q, et al. Effects of long-term coated sodium butyrate supplementation on the intestinal health and colonization of cecal *Salmonella* of laying hens infected with *Salmonella enteritidis*. Animals. 2024;14(9):1356. 10.3390/ani14091356. 38731359 10.3390/ani14091356 PMC11083467

[16] Hou Q, Li G, Pan X, Zhong X, Geng X, Yang X, et al. Long-term supplementation of genistein improves immune homeostasis in the aged gut and extends the laying cycle of aged laying hens. Poult Sci. 2024. 10.1016/j.psj.2024.103670. 10.1016/j.psj.2024.103670 PMC11017059 38598909

[17] Chen X, Wang Y, Chen Y, Dai J, Cheng S, Chen X. Formation, physicochemical properties, and biological activities of theabrownins. Food Chem. 2024;448:139140. 10.1016/j.foodchem.2024.139140. 38574720 10.1016/j.foodchem.2024.139140

[18] Xiao Y, Li M, Wu Y, Zhong K, Gao H. Structural characteristics and hypolipidemic activity of theabrownins from dark tea fermented by single species *Eurotium cristatum* PW-1. Biomolecules. 2020. 10.3390/biom10020204. 10.3390/biom10020204 PMC7072556 32019226

[19] Zhang Y, Ying M, Wang Z, Feng W, Lu X, Zhou W, et al. Physicochemical characterization of the fraction components of the theabrownin isolates from Pu’er tea. LWT. 2024;200:116147. 10.1016/j.lwt.2024.116147.

[20] Cai Y, Huang Y, Qiu L, Mi X, Wang Y, Tao X, et al. Theabrownins prevents DSS-induced colitis via modulating PPAR-γ and NF-κB signaling pathways in mice. J Funct Foods. 2023;109:105812. 10.1016/j.jff.2023.105812.

[21] Leung HKM, Lo EKK, El-Nezami H. Theabrownin alleviates colorectal tumorigenesis in murine AOM/DSS model via PI3K/Akt/mTOR pathway suppression and gut microbiota modulation. Antioxidants. 2022;11(9):1716. 10.3390/antiox11091716. 36139789 10.3390/antiox11091716 PMC9495753

[22] Lei S, Zhang Z, Xie G, Zhao C, Miao Y, Chen D, et al. Theabrownin modulates the gut microbiome and serum metabolome in aging mice induced by D-galactose. J Funct Foods. 2022;89:104941. 10.1016/j.jff.2022.104941.

[23] Wang J, Zhang T, Wan C, Lai Z, Li J, Chen L, et al. The effect of theabrownins on the amino acid composition and antioxidant properties of hen eggs. Poult Sci. 2023;102(11):102717. 10.1016/j.psj.2023.102717. 37734359 10.1016/j.psj.2023.102717 PMC10518584

[24] Xiao Y, Yang D, Zhang H, Guo H, Liao Y, Lian C, et al. Theabrownin as a potential prebiotic compound regulates lipid metabolism via the gut microbiota, microbiota-derived metabolites, and hepatic FoxO/PPAR signaling pathways. J Agric Food Chem. 2024. 10.1021/acs.jafc.3c08541. 38567990 10.1021/acs.jafc.3c08541

[25] Yang W, Ren D, Shao H, Zhang X, Li T, Zhang L, et al. Theabrownin from Fu Brick tea improves ulcerative colitis by shaping the gut microbiota and modulating the tryptophan metabolism. J Agric Food Chem. 2023. 10.1021/acs.jafc.2c06821. 36728562 10.1021/acs.jafc.2c06821

[26] Xu W, Ayu Y, Wang J, Zeng Q, Bai S, Ding X, et al. Effects of dietary theabrownins on production performance, egg quality, and ovarian function of laying hens with different ages. Poult Sci. 2023;102(6):102545. 10.1016/j.psj.2023.102545. 37019071 10.1016/j.psj.2023.102545 PMC10106962

[27] National Research Council. Nutrient requirements of poultry. 9th rev. ed. Washington, DC: National Academy Press; 1994.

[28] Ministry of Agriculture and Rural Affairs of the People’s Republic of China. Feeding Standard of Chicken (NY/T 33–2004). Beijing: China Agriculture Press; 2004.

[29] Gu YF, Chen YP, Jin R, Wang C, Wen C, Zhou YM. A comparison of intestinal integrity, digestive function, and egg quality in laying hens with different ages. Poult Sci. 2021;100(3):100949. 10.1016/j.psj.2020.12.046. 33652523 10.1016/j.psj.2020.12.046 PMC7936206

[30] Liu T, Li C, Li Y, Feng F. Glycerol monolaurate enhances reproductive performance, egg quality and albumen amino acids composition in aged hens with gut microbiota alternation. Agriculture. 2020;10(7):250. 10.3390/agriculture10070250.

[31] Gan L, Zhao Y, Mahmood T, Guo Y. Effects of dietary vitamins supplementation level on the production performance and intestinal microbiota of aged laying hens. Poult Sci. 2020;99(7):3594–605. 10.1016/j.psj.2020.04.007. 32616256 10.1016/j.psj.2020.04.007 PMC7597815

[32] Alfonso-Carrillo C, Benavides-Reyes C, De Los MJ, Dominguez-Gasca N, Sanchez-Rodríguez E, Garcia-Ruiz AI, et al. Relationship between bone quality, egg production and eggshell quality in laying hens at the end of an extended production cycle (105 weeks). Animals. 2021;11(3):623. 10.3390/ani11030623. 10.3390/ani11030623 PMC7996911 33652961

[33] Chang X, Wang B, Zhang H, Qiu K, Wu S. The change of albumen quality during the laying cycle and its potential physiological and molecular basis of laying hens. Poult Sci. 2024;103(10):104004. 10.1016/j.psj.2024.104004. 39067125 10.1016/j.psj.2024.104004 PMC11331942

[34] Zhang T, Bai S, Ding X, Zeng Q, Zhang K, Lv L, et al. Dietary theabrownin supplementation improves production performance and egg quality by promoting intestinal health and antioxidant capacity in laying hens. Animals. 2022;12(20):2856. 10.3390/ani12202856. 36290242 10.3390/ani12202856 PMC9597818

[35] Yang JH, Zhao ZH, Hou JF, Zhou ZL, Deng YF, Dai JJ. Expression of TRPV6 and CaBP-D28k in the egg shell gland (uterus) during the oviposition cycle of the laying hen. Br Poult Sci. 2013;54(3):398–406. 10.1080/00071668.2013.791385. 23796121 10.1080/00071668.2013.791385

[36] Huang F, Zheng X, Ma X, Jiang R, Zhou W, Zhou S, et al. Theabrownin from Pu-erh tea attenuates hypercholesterolemia via modulation of gut microbiota and bile acid metabolism. Nat Commun. 2019;10:4971. 10.1038/s41467-019-12896-x. 10.1038/s41467-019-12896-x PMC6823360 31672964

[37] Yu X, Li J, Peng R, Zhang X, Yue W, Wang Y, et al. Flos lonicerae and baikal skullcap extracts improved laying performance of aged hens partly by modulating antioxidant capacity, immune function, cecal microbiota and ovarian metabolites. Animals. 2025;15(19):2882. 10.3390/ani15192882. 41096477 10.3390/ani15192882 PMC12523767

[38] Nguyen D. Relationship between the ratio of villous height:crypt depth and gut bacteria counts as well production parameters in broiler chickens. J Agric Dev. 2021;20:1–10. 10.52997/jad.1.03.2021.

[39] Sun Y, Li Z, Yan M, Zhao H, He Z, Zhu M. Responses of intestinal antioxidant capacity, morphology, barrier function, immunity, and microbial diversity to chlorogenic acid in late-peak laying hens. Animals. 2024;14(20):2957. 10.3390/ani14202957. 10.3390/ani14202957 PMC11503754 39457887

[40] Yegani M, Korver DR. Factors affecting intestinal health in poultry. Poult Sci. 2008;87(10):2052–63. 10.3382/ps.2008-00091. 18809868 10.3382/ps.2008-00091 PMC7107194

[41] Zhang T, Bai S, Ding X, Zeng Q, Xuan Y, Xu S, et al. Pu-erh tea theabrownin improves the ovarian function and gut microbiota in laying hens. Poult Sci. 2024;103(7):103795. 10.1016/j.psj.2024.103795. 38723460 10.1016/j.psj.2024.103795 PMC11101868

[42] Tan BL, Norhaizan ME, Liew WPP, Sulaiman Rahman H. Antioxidant and oxidative stress: a mutual interplay in age-related diseases. Front Pharmacol. 2018;9:1162. 10.3389/fphar.2018.01162. 10.3389/fphar.2018.01162 PMC6204759 30405405

[43] Wan MLY, Ling KH, Wang MF, El-Nezami H. Green tea polyphenol epigallocatechin-3-gallate improves epithelial barrier function by inducing the production of antimicrobial peptide pBD-1 and pBD-2 in monolayers of porcine intestinal epithelial IPEC-J2 cells. Mol Nutr Food Res. 2016;60(5):1048–58. 10.1002/mnfr.201500992. 26991948 10.1002/mnfr.201500992

[44] Xu Y, Yin F, Wang J, Wu P, Qiu X, He X, et al. Effect of tea polyphenols on intestinal barrier and immune function in weaned lambs. Front Vet Sci. 2024;11:1361507. 10.3389/fvets.2024.1361507. 38435366 10.3389/fvets.2024.1361507 PMC10904598

[45] Pisoschi AM, Pop A. The role of antioxidants in the chemistry of oxidative stress: A review. Eur J Med Chem. 2015;97:55–74. 10.1016/j.ejmech.2015.04.040. 25942353 10.1016/j.ejmech.2015.04.040

[46] Inumaru J, Nagano O, Takahashi E, Ishimoto T, Nakamura S, Suzuki Y, et al. Molecular mechanisms regulating dissociation of cell-cell junction of epithelial cells by oxidative stress. Genes Cells. 2009;14(6):703–16. 10.1111/j.1365-2443.2009.01303.x. 19422420 10.1111/j.1365-2443.2009.01303.x

[47] Finkel T, Holbrook NJ. Oxidants, oxidative stress and the biology of ageing. Nature. 2000;408(6809):239–47. 10.1038/35041687. 11089981 10.1038/35041687

[48] Xian Y, Da P, Chao Y, Hui X, Ligang Y, Shaokang W, et al. Wheat oligopeptides enhance the intestinal mucosal barrier and alleviate inflammation via the TLR4/Myd88/MAPK signaling pathway in aged mice. Food Nutr Res. 2022. 10.29219/fnr.v66.5690. 10.29219/fnr.v66.5690 PMC8861859 35261579

[49] Zou Y, Yan H, Li C, Wen F, Jize X, Zhang C, et al. A pectic polysaccharide from *Codonopsis pilosula* alleviates inflammatory response and oxidative stress of aging mice via modulating intestinal microbiota-related gut–liver axis. Antioxidants. 2023;12(9):1781. 10.3390/antiox12091781. 37760084 10.3390/antiox12091781 PMC10525188

[50] Zeng J, Deng Z, Zou Y, Liu C, Fu H, Gu Y, et al. Theaflavin alleviates oxidative injury and atherosclerosis progress via activating microRNA-24-mediated Nrf2/HO-1 signal. Phytother Res. 2021;35(6):3418–27. 10.1002/ptr.7064. 33755271 10.1002/ptr.7064

[51] Scalbert A, Manach C, Morand C, Rémésy C, Jiménez L. Dietary polyphenols and the prevention of diseases. Crit Rev Food Sci Nutr. 2005;45(4):287–306. 10.1080/1040869059096. 16047496 10.1080/1040869059096

[52] Wang S, Yang H. Low-molecular-weight heparin ameliorates intestinal barrier dysfunction in aged male rats via protection of tight junction proteins. Biogerontology. 2024;25(6):1039–51. 10.1007/s10522-024-10118-6. 38970715 10.1007/s10522-024-10118-6

[53] Li Y, Liu J, Pongkorpsakol P, Xiong Z, Li L, Jiang X, et al. Relief effects of icariin on inflammation-induced decrease of tight junctions in intestinal epithelial cells. Front Pharmacol. 2022;13:903762. 10.3389/fphar.2022.903762. 35754510 10.3389/fphar.2022.903762 PMC9214228

[54] Ren WY, Wu KF, Li X, Luo M, Liu HC, Zhang SC, et al. Age-related changes in small intestinal mucosa epithelium architecture and epithelial tight junction in rat models. Aging Clin Exp Res. 2014;26(2):183–91. 10.1007/s40520-013-0148-0. 10.1007/s40520-013-0148-0 24243034

[55] Ma TY, Boivin MA, Ye D, Pedram A, Said HM. Mechanism of TNF-α modulation of Caco-2 intestinal epithelial tight junction barrier: role of myosin light-chain kinase protein expression. Am J Physiol Gastrointest Liver Physiol. 2005;288(3):G422–30. 15701621 10.1152/ajpgi.00412.2004

[56] Watari A, Sakamoto Y, Hisaie K, Iwamoto K, Fueta M, Yagi K, et al. Rebeccamycin attenuates TNF-α-induced intestinal epithelial barrier dysfunction by inhibiting myosin light chain kinase production. Cell Physiol Biochem. 2017;41(5):1924–34. 10.1159/000472367. 28391269 10.1159/000472367

[57] He F, Peng J, Deng XL, Yang LF, Camara AD, Omran A, et al. Mechanisms of tumor necrosis factor-alpha-induced leaks in intestine epithelial barrier. Cytokine. 2012;59(2):264–72. 10.1016/j.cyto.2012.04.008. 10.1016/j.cyto.2012.04.008 22583690

[58] Lan J, Dou X, Li J, Yang Y, Xue C, Wang C, et al. L-Arginine ameliorates lipopolysaccharide-induced intestinal inflammation through inhibiting the TLR4/NF-κB and MAPK pathways and stimulating β-defensin expression in vivo and in vitro. J Agric Food Chem. 2020;68(9):2648–63. 10.1021/acs.jafc.9b07611. 32064872 10.1021/acs.jafc.9b07611

[59] Zhao L, Zhao C, Miao Y, Lei S, Li Y, Gong J, et al. Theabrownin from Pu-erh tea improves DSS-induced colitis via restoring gut homeostasis and inhibiting TLR2&4 signaling pathway. Phytomedicine. 2024;132:155852. 10.1016/j.phymed.2024.155852. 39029137 10.1016/j.phymed.2024.155852

[60] Yoo JY, Groer M, Dutra SVO, Sarkar A, Mcskimming DI. Gut microbiota and immune system interactions. Microorganisms. 2020. 10.3390/microorganisms8101587. 10.3390/microorganisms8101587 PMC7602490 33076307

[61] Iacob S, Iacob DG. Infectious threats, the intestinal barrier, and its trojan horse: dysbiosis. Front Microbiol. 2019;10:1676. 10.3389/fmicb.2019.01676. 10.3389/fmicb.2019.01676 PMC6692454 31447793

[62] Xu Y, Yang H, Zhang L, Su Y, Shi D, Xiao H, et al. High-throughput sequencing technology to reveal the composition and function of cecal microbiota in Dagu chicken. BMC Microbiol. 2016;16:259. 10.1186/s12866-016-0877-2. 10.1186/s12866-016-0877-2 PMC5097418 27814685

[63] Singh P, Karimi A, Devendra K, Waldroup PW, Cho KK, Kwon YM. Influence of penicillin on microbial diversity of the cecal microbiota in broiler chickens. Poult Sci. 2013;92(1):272–6. 10.3382/ps.2012-02603. 23243258 10.3382/ps.2012-02603

[64] Vasilopoulos S, Giannenas I, Mellidou I, Stylianaki I, Antonopoulou E, Tzora A, et al. Diet replacement with whole insect larvae affects intestinal morphology and microbiota of broiler chickens. Sci Rep. 2024;14:6836. 10.1038/s41598-024-54184-9. 10.1038/s41598-024-54184-9 PMC10957974 38514719

[65] Elokil AA, Chen W, Mahrose K, Elattrouny MM, Abouelezz KFM, Ahmad HI, et al. Early life microbiota transplantation from highly feed-efficient broiler improved weight gain by reshaping the gut microbiota in laying chicken. Front Microbiol. 2022;13:1022783. 10.3389/fmicb.2022.1022783. 36466637 10.3389/fmicb.2022.1022783 PMC9715608

[66] Emami NK, Calik A, White MB, Kimminau EA, Dalloul RA. Effect of probiotics and multi-component feed additives on microbiota, gut barrier and immune responses in broiler chickens during subclinical necrotic enteritis. Front Vet Sci. 2020;7:572142. 10.3389/fvets.2020.572142. 33324697 10.3389/fvets.2020.572142 PMC7725796

[67] Parkar SG, Trower TM, Stevenson DE. Fecal microbial metabolism of polyphenols and its effects on human gut microbiota. Anaerobe. 2013;23:12–9. 10.1016/j.anaerobe.2013.07.009. 23916722 10.1016/j.anaerobe.2013.07.009

[68] Sun Y, Zhang S, Nie Q, He H, Tan H, Geng F, et al. Gut firmicutes: Relationship with dietary fiber and role in host homeostasis. Crit Rev Food Sci Nutr. 2023;63(33):12073–88. 10.1080/10408398.2022.2098249. 35822206 10.1080/10408398.2022.2098249

[69] Niu J, Zhang J, Wei L, Ma X, Zhang W, Nie C. Cottonseed meal fermented by *Candida tropical* reduces the fat deposition in white-feather broilers through cecum bacteria-host metabolic cross-talk. Appl Microbiol Biotechnol. 2020;104(10):4345–57. 10.1007/s00253-020-10538-7. 32232527 10.1007/s00253-020-10538-7

[70] Gao Y, Davis B, Zhu W, Zheng N, Meng D, Walker WA. Short-chain fatty acid butyrate, a breast milk metabolite, enhances immature intestinal barrier function genes in response to inflammation in vitro and in vivo. Am J Physiol Gastrointest Liver Physiol. 2021;320(4):G521–30. 10.1152/ajpgi.00279.2020. 33085904 10.1152/ajpgi.00279.2020 PMC8238162

[71] Matheus VA, Oliveira RB, Maschio DA, Tada SFS, Soares GM, Mousovich-Neto F, et al. Butyrate restores the fat/lean mass ratio balance and energy metabolism and reinforces the tight junction-mediated intestinal epithelial barrier in prediabetic mice independently of its anti-inflammatory and epigenetic actions. J Nutr Biochem. 2023;120:109409. 10.1016/j.jnutbio.2023.109409. 37364792 10.1016/j.jnutbio.2023.109409

[72] Derrien M, Van Baarlen P, Hooiveld G, Norin E, Müller M, De Vos WM. Modulation of mucosal immune response, tolerance, and proliferation in mice colonized by the mucin-degrader *Akkermansia muciniphila*. Front Microbiol. 2011;2:166. 10.3389/fmicb.2011.00166. 21904534 10.3389/fmicb.2011.00166 PMC3153965

[73] Ottman N, Geerlings SY, Aalvink S, De Vos WM, Belzer C. Action and function of *Akkermansia muciniphila* in microbiome ecology, health and disease. Best Pract Res Clin Gastroenterol. 2017;31(6):637–42. 10.1016/j.bpg.2017.10.001. 29566906 10.1016/j.bpg.2017.10.001

[74] Liu MJ, Yang JY, Yan ZH, Hu S, Li JQ, Xu ZX, et al. Recent findings in *Akkermansia muciniphila*-regulated metabolism and its role in intestinal diseases. Clin Nutr. 2022;41(10):2333–44. 10.1016/j.clnu.2022.08.029. 36113229 10.1016/j.clnu.2022.08.029

[75] Wang Z, Li B, Bao L, Chen Y, Yang J, Xu F, et al. *Fusobacterium nucleatum* aggravates intestinal barrier impairment and colitis through IL-8 induced neutrophil chemotaxis by activating epithelial cells. J Inflamm Res. 2024;17:8407–20. 10.2147/JIR.S470376. 39534061 10.2147/JIR.S470376 PMC11556331

[76] Zhang M, Zhou L, Wang Y, Dorfman RG, Tang D, Xu L, et al. *Faecalibacterium prausnitzii* produces butyrate to decrease c-Myc-related metabolism and Th17 differentiation by inhibiting histone deacetylase 3. Int Immunol. 2019;31(8):499–514. 10.1093/intimm/dxz022. 30809639 10.1093/intimm/dxz022

[77] Ferreira-Halder CV, Faria AVdS, Andrade SS. Action and function of *Faecalibacterium prausnitzii* in health and disease. Best Prac Res Clin Gastroenterol. 2017;31(6):643–8. 10.1016/j.bpg.2017.09.011. 10.1016/j.bpg.2017.09.011 29566907

[78] Ahmadi A, Kouhsari E, Razavi S, Mohamadzadeh N, Besharat S, Vakili MA, et al. Comparative analysis of dominant gut microbiota in Inflammatory Bowel Disease patients and healthy individuals: A case-control study. New Microbes New Infect. 2025;64:101567. 10.1016/j.nmni.2025.101567. 39991465 10.1016/j.nmni.2025.101567 PMC11846925

[79] Jin DX, Jia CY, Yang B, Wu YH, Chen L, Liu R, et al. The ameliorative mechanism of *Lactiplantibacillus plantarum* NJAU-01 against D-galactose induced oxidative stress: a hepatic proteomics and gut microbiota analysis. Food Funct. 2024;15(11):6174–88. 10.1039/D4FO00406J. 38770619 10.1039/d4fo00406j

[80] Hashizume K, Tsukahara T, Yamada K, Koyama H, Ushida K. *Megasphaera elsdenii* JCM1772T normalizes hyperlactate production in the large intestine of fructooligosaccharide-fed rats by stimulating butyrate production. J Nutr. 2003;133(10):3187–90. 10.1093/jn/133.10.3187. 10.1093/jn/133.10.3187 14519808

[81] Zhen Y, Xi Z, Nasr SM, He F, Han M, Yin J, et al. Multi-omics reveals the impact of exogenous short-chain fatty acid infusion on rumen homeostasis: insights into crosstalk between the microbiome and the epithelium in a goat model. Microbiol Spectr. 2023;11(4):e0534322. 10.1128/spectrum.05343-22. 37439665 10.1128/spectrum.05343-22 PMC10433986

[82] Al-Sadi R, Nighot P, Nighot M, Haque M, Rawat M, Ma TY. *Lactobacillus acidophilus* induces a strain-specific and Toll-Like receptor 2-dependent enhancement of intestinal epithelial tight junction barrier and protection against intestinal inflammation. Am J Pathol. 2021;191(5):872–84. 10.1016/j.ajpath.2021.02.003. 33607043 10.1016/j.ajpath.2021.02.003 PMC8110190

[83] Mujagic Z, De Vos P, Boekschoten MV, Govers C, Pieters HH, De Wit NJ, et al. The effects of *Lactobacillus plantarum* on small intestinal barrier function and mucosal gene transcription; a randomized double-blind placebo controlled trial. Sci Rep. 2017;7:40128. 10.1038/srep40128. 28045137 10.1038/srep40128 PMC5206730

[84] Chen X, Hu Y, Wang B, Chen Y, Yuan Y, Zhou W, et al. Characterization of theabrownins prepared from tea polyphenols by enzymatic and chemical oxidation and their inhibitory effect on colon cancer cells. Front Nutr. 2022;9:849728. 10.3389/fnut.2022.849728. 35369086 10.3389/fnut.2022.849728 PMC8965357

