# Supplementary material for: Dietary theabrownins alleviate age-related declines in productive performance and egg quality by improving intestinal health in laying hens
Source: J Anim Sci Biotechnol. 2026 Aug 10;17:162. doi: 10.1186/s40104-026-01480-1 (PMC13455484; doi:10.1186/s40104-026-01480-1)
Supplement: Supplementary file 1 [file 40104_2026_1480_MOESM1_ESM.docx]

**Fig S1.** Weekly dynamic changes in productive performance during the 12-week experimental period. (A) Laying rate; (B) egg weight; (C) egg mass; (D) feed-to-egg ratio; (E) average daily feed intake (ADFI); and (F) unqualified egg rate. Values are presented as means ± SEM. P-values were obtained from a mixed model including age, TB supplementation, week, and their interactions as fixed effects. *n* =8.
